# Ocrelizumab transiently alters microbiota and modulates immune response depending on treatment outcome

**DOI:** 10.1016/j.isci.2025.113872

**Published:** 2025-10-31

**Authors:** Stepan Coufal, Zuzana Jiraskova Zakostelska, Tomas Thon, Radka Roubalova, Dominika Kadleckova, Martina Salakova, Ruth Tachezy, Tomas Hrncir, Miloslav Kverka, Veronika Ticha, Miluse Pavelcova, Pavlina Kleinova, Jana Lizrova Preiningerova, Ivana Kovarova, Jakub Kreisinger, Helena Tlaskalova-Hogenova, Eva Kubala Havrdova

**Affiliations:** 1 Laboratory of Cellular and Molecular Immunology, Institute of Microbiology of the Czech Academy of Sciences, Prague, Czech Republic; 2 Department of Genetics and Microbiology, Faculty of Science, Charles University, BIOCEV, Vestec, Czech Republic; 3 Laboratory of Gnotobiology, Institute of Microbiology of the Czech Academy of Sciences, Novy Hradek, Czech Republic; 4 Department of Neurology and Centre of Clinical Neuroscience, First Medical Faculty, Charles University and General Medical Hospital in Prague, Prague, Czech Republic; 5 Laboratory of Animal Evolutionary Biology, Faculty of Science, Department of Zoology, Charles University, Prague, Czech Republic

**Keywords:** immune response, microbiome, neuroscience

## Abstract

Multiple sclerosis (MS) is an autoimmune disease characterized by central nervous system atrophy. Microbiota dysbiosis is implicated in MS pathogenesis and treatment outcomes. In our study, we observed microbiota changes already present in treatment-naïve individuals with clinically isolated syndrome, affecting both bacteria and viruses. Gut bacteria alterations were transient during the first 12 months of anti-CD20 therapy. After 12 months, responders showed increased gut microbiota alpha diversity approaching healthy control levels, while non-responders showed a significant decline. Key changes involved *Parabacteroides* spp., producers of short-chain fatty acids that support gut barrier function and have anti-inflammatory potential. We detected altered gut barrier biomarkers and antibodies against common commensals in MS patients, which were modulated by anti-CD20 treatment. Notably, lipopolysaccharide-binding protein and mannose-binding lectin decreased only in responders. These findings suggest that intestinal barrier damage contributes to immune responses linked to microbial translocation, MS pathogenesis, and treatment outcomes.

## Highlights

• Individuals with clinically isolated syndrome have gut microbiota dysbiosis
• Alpha diversity decreases during treatment only in non-responders
• Gut barrier disruption causes microbial translocation and low-grade inflammation
• Short-chain fatty acid producers are linked to treatment outcome

## Graphical abstract

**Figure:**
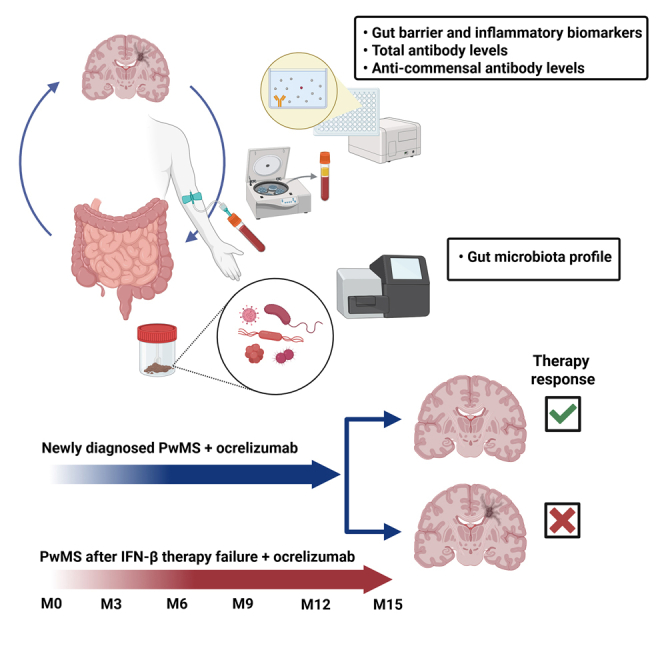

## Introduction

Multiple sclerosis (MS) is an autoimmune demyelinating disease of the central nervous system (CNS) that is usually diagnosed in young adults. If untreated, it leads to severe disability within 15 years. The loss of myelin caused by inflammation and axonal damage leads to CNS atrophy. MS is triggered by a combination of factors, with the genetic component accounting for less than a third of the risk. Other aspects such as hypovitaminosis D, previous Epstein-Barr virus (EBV) infections, smoking, and obesity have also been identified as possible risk factors.^1^ In recent years, a link between the microbiota and the immune system has been discovered and identified as an important pathogenic factor in autoimmune diseases outside the gut.^2^ Preclinical studies using animal models of autoimmune diseases and germ-free conditions have shown that gut microbiota plays a key role in disease development.^3^ The widely used experimental autoimmune encephalomyelitis (EAE) model was used to confirm the involvement of the microbiota in the pathogenesis of MS.^4^^,^^5^ The gut microbiota of persons with MS (PwMS) significantly differs from healthy controls (HCs), suggesting that the gut microbiome plays a role in the development of MS.^6^ However, whether the dysbiosis is the cause or a consequence of autoimmune diseases such as MS remains unanswered.^7^

In addition, certain viruses such as cytomegalovirus, varicella zoster virus, and EBV have been linked to the development of MS and modulation of the immune system. Infection with EBV significantly increases the risk of developing MS later in life and even often immediately precedes the development of the disease.^8^ EBV could affect the CNS through a mechanism called molecular mimicry, which may be mediated between nuclear factor antigen 1 and the CNS protein glial cell adhesion molecule.^9^ This supports the finding that EBV-infected auto-reactive B cells can accumulate in the CNS in response to impaired cytotoxic CD8^+^ T cell immunity.^10^ There is no cure for MS; current treatment focuses on managing the disease and its symptoms. Biologicals such as ocrelizumab are now the cornestone of disease-modifying therapies. Ocrelizumab, a recombinant humanized monoclonal antibody against the CD20, is a generally well-tolerated and a highly effective treatment option for MS.^11^ Since it is able to both prevent relapses and slow down the progression of disability, it has been approved for relapsing-remitting, active secondary progressive, and primary progressive MS.^11^^,^^12^^,^^13^

The efficacy of treatment of MS with disease-modifying drugs (DMDs) is defined by achieving NEDA (no evidence of disease activity) status, i.e., no evidence of clinical activity (such as relapses, worsening disability) or radiologic evidence of disease activity (e.g., new or enlarging lesions on brain magnetic resonance imaging).^2^^,^^3^ PwMS who do not achieve this status are considered non-responders to the specific treatment and receive alternative treatments. Treatment efficacy varies widely from patient to patient, and there are no known predictors of treatment response. Despite many new effective treatment options with different mechanisms of action, satisfactory efficacy (i.e., NEDA status) is achieved in less than half of PwMS.

Microbiota changes have recently been observed in PwMS patients treated with glatiramer acetate and dimethyl fumarate, both of which were found to alter gut microbiome composition and influence key metabolic pathways.^14^ Although the significance of this finding is not yet known, further investigation into the role of the gut microbiota on the pathogenesis and treatment of MS is needed. Early studies of the microbiota in PwMS showed a tendency toward “normalization” of the microbiota in PwMS on DMD treatment. While we have preliminary evidence of changes in the composition of the fecal microbiota associated with interferon beta (IFN-β), glatiramer acetate, or fumaric acid treatment, there is limited data for other DMDs, including monoclonal antibodies such as ocrelizumab. In addition, there are no studies describing how the microbiota is related to treatment efficacy in individual PwMS or whether the microbiota is a factor affecting response or non-response to a particular treatment.

Current data clearly show that the commensal gut microbiota can tune the inflammatory response of the immune system through multiple interlinked mechanisms, such that it can influence inflammation not only in the gut but also in distant tissues such as the CNS.^3^ Given the immunomodulatory nature of current MS treatment, the microbiota could be a suitable biomarker for disease progression or pharmacological response to MS treatments. Therefore, the aim of the present study was to describe changes in the microbiota of PwMS treated with ocrelizumab over 12 months. Given the complexity of MS pathogenesis, we linked these changes to the serum levels of anti-commensal antibodies and molecules associated with intestinal barrier damage and inflammatory response and regulation. Moreover, the changes in the composition of the virome were also evaluated. We assessed if these changes were related to treatment response with the goal of identifying potential candidates associated with treatment outcomes.

## Results

### Alpha diversity of the gut microbiota transiently decreased for six months after the start of ocrelizumab treatment

First, we confirmed that newly diagnosed PwMS have lower alpha diversity compared to HCs based on the Shannon diversity, observed amplicon sequence variants (ASVs), and Chao1 indices. The alpha diversity was lower to HC at M0 and it further slightly decreased after three months (M3) of anti-CD20 treatment and reached its significantly lowest level after month six (M6).

**Figure 1:**
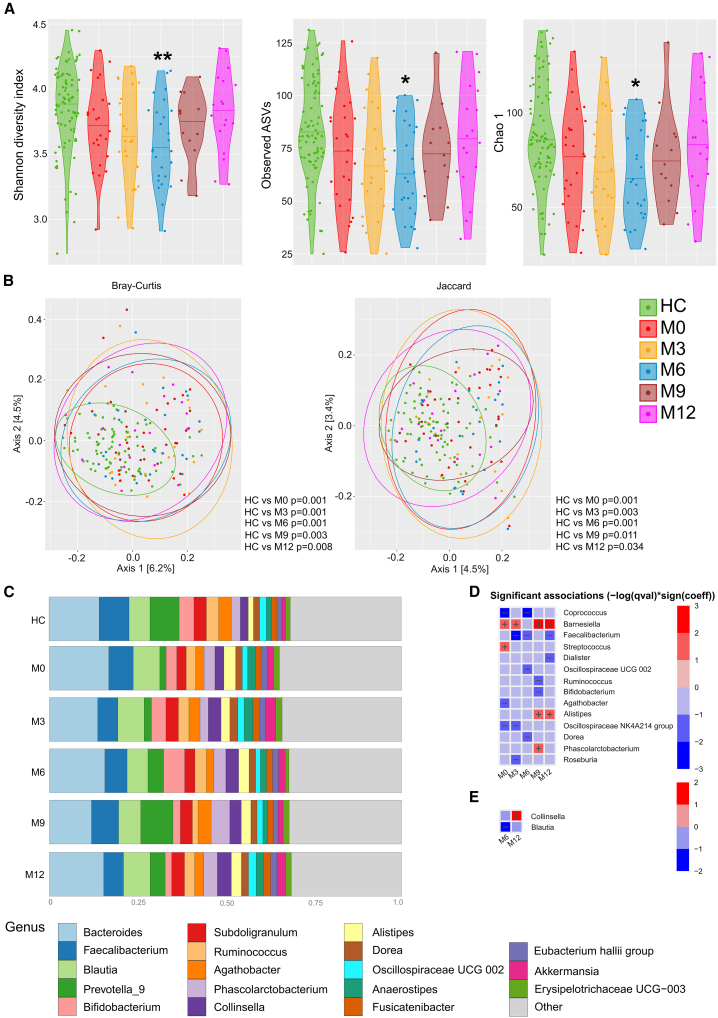
Gut microbiota of newly diagnosed PwMS treated with ocrelizumab as a first-line therapy Alpha diversity is expressed using the Shannon diversity index, Observed ASVs, and Chao1; data are presented as median (A). Beta diversity is expressed using the Bray-Curtis and Jaccard index (B). Bar plot of the 18 most abundant bacterial taxa (C) and heatmap with significant associations of genera with treatment duration (months), compared to healthy controls (HC) (D) or to M0 (E). ∗ (*p* < 0.05) or ∗∗ (*p* < 0.01); HC *n* = 81, M0 *n* = 25, M3 *n* = 23, M6 *n* = 26, M9 *n* = 13, and M12 *n* = 18.

After this point, alpha diversity slowly began to increase (M9), reaching levels similar to HCs by the end of month 12 (M12) (Figure 1A). We observed the same patterns for all three alpha diversity indices (Figure 1A). Analysis of beta diversity showed that newly diagnosed PwMS had a significantly different beta diversity at baseline (M0), measured by both the Bray-Curtis and Jaccard dissimilarity indices, compared to HCs. This difference remained significant throughout treatment (Figures 1B and S1). We did not observe significant changes in beta diversity in newly diagnosed PwMS undergoing treatment (M0–M12).

The relative abundances of the 18 most abundant taxa are plotted across months of ocrelizumab treatment and in HCs (Figure 1C). When compared to HCs, newly diagnosed PwMS had a lower representation of genera *Coprococcus*, *Agathobacter*, and *Oscillospiraceae* of the NK4A214 group and a higher representation of genera *Streptococcus* and *Barnesiella* at M0 and a lower representation of genera *Faecalibacterium* and *Roseburia* after three months of treatment (M3) (Figure 1D). During treatment, lower numbers of *Blautia* spp. were found at the end of month six (M6), and higher numbers of *Collinsella* spp. at the end of month 12 (M12) compared to PwMS before treatment (M0) (Figures 1E and S2).

**Figure 2:**
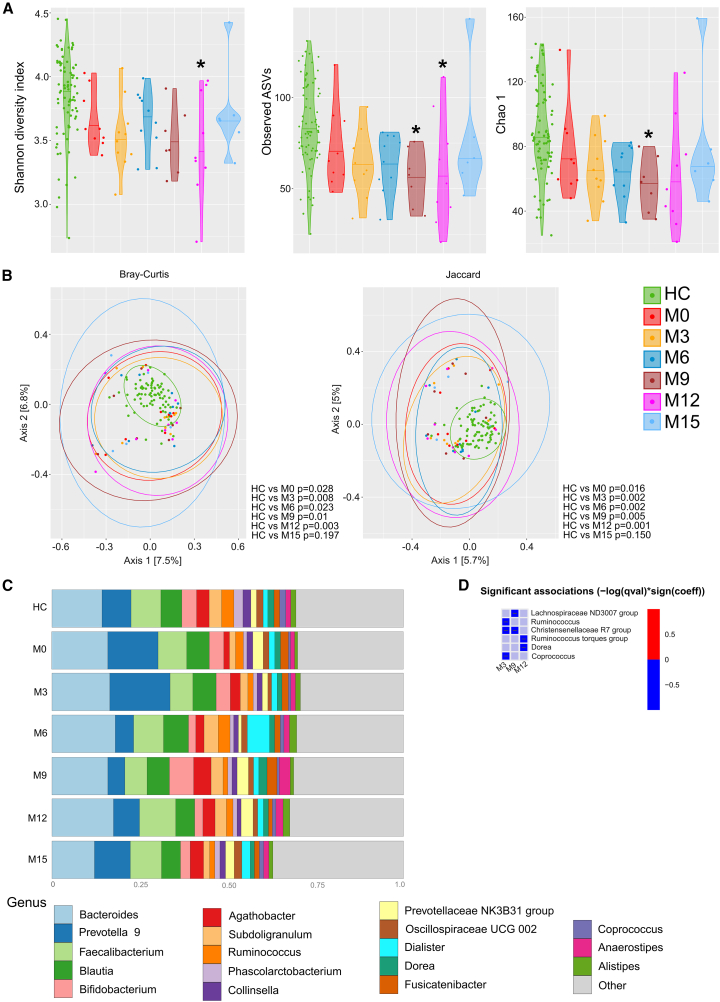
Gut microbiota of PwMS who previously relapsed on IFN-β treatment treated with ocrelizumab Alpha diversity is expressed as the Shannon diversity index, Observed ASVs, and Chao1; data are presented as median (A). Beta diversity is expressed as the Bray-Curtis and Jaccard index (B). Bar plot of the 18 most abundant bacterial taxa (C) and a heatmap with significant associations of genera with treatment duration (months), compared to HC (D). ∗ (*p* < 0.05); HC *n* = 81, M0 *n* = 9, M3 *n* = 10, M6 *n* = 10, M9 *n* = 7, M12 *n* = 9, and M15 *n* = 6.

To determine whether these changes in the gut microbiota were specific to ocrelizumab-treated PwMS without prior therapy, we also analyzed the changes in the gut microbiota of PwMS who switched to ocrelizumab treatment after relapse on IFN-β treatment. Interestingly, we also found a similar pattern of alpha diversity indices in these PwMS (Figure 2A). The only difference was that the transient decrease in alpha diversity was postponed to a later time point, reaching the lowest value at M12, before increasing again at M15. In some of these PwMS, we were able to analyze the gut microbiota at M18, 21, and 24. After M15, the alpha diversity did not change significantly, and by the M24, alpha diversity was similar to HCs (data not shown). Thus, ocrelizumab treatment resulted in a transient decrease in alpha diversity in both treatment-naïve patients and patients with a history of IFN-β treatment, although this trend shifted to the later time point in the latter cohort. Similarly, in patients treated with ocrelizumab after IFN-β treatment failure the beta diversity was shifted between M3 and M12; however, at M15, the gut microbiota resembles that of HC (Figures 2B and S3). We found fluctuation in the genus *Prevotella* abundance with the highest abundance at M0–M3 and lowest at M6–M9 (Figure 2C). And although neither one was significant, *Prevotella* abundance did not stay constant during the observed period. However, compared to HCs, PwMS had significantly decreased abundance of genera *Ruminococcus*, *Christensenellaceae* group R7, and *Coprococcus* at M3, *Lachnospiraceae* ND3008 at M9, and *Ruminococcus torques* and *Dorea* at M12. There were no significant differences when comparing treatment months with M0 (Figure 2D).

### Virome diversity and composition shift during ocrelizumab treatment

**Figure 3:**
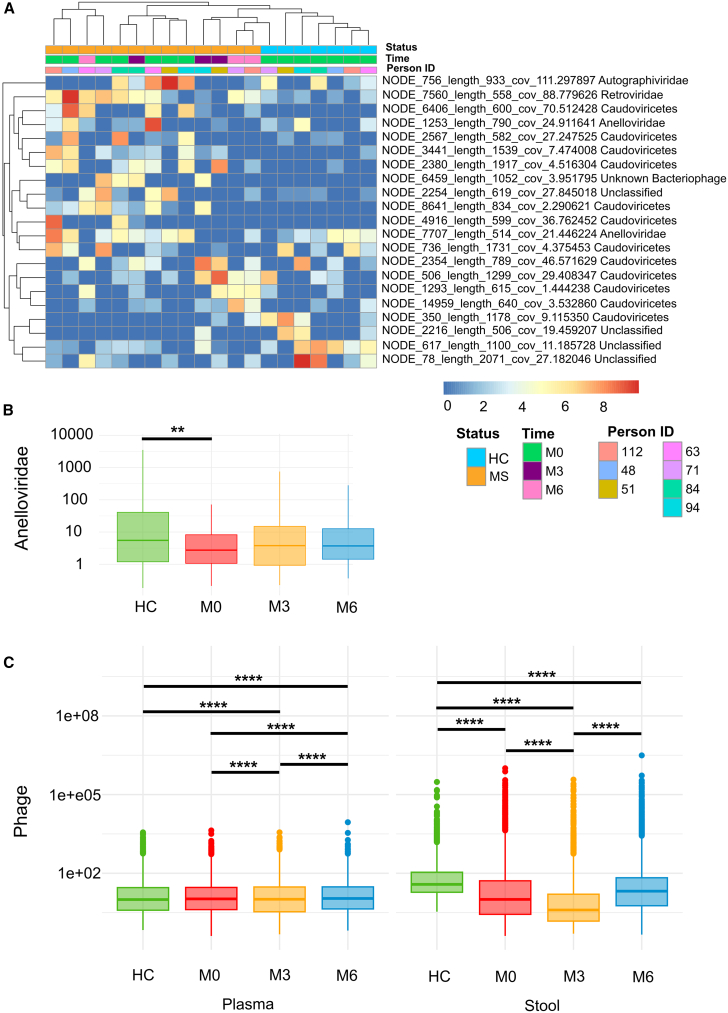
Virome profile of PwMS treated with ocrelizumab as the first-line therapy and HC from the same household Heatmap of differentially abundant viral contigs in plasma with significantly differentially abundant viral contigs identified using the DESeq2 (*p* < 0.05) (A). Relative abundance of anelloviruses, each dot represents the detection of an individual anellovirus species in a single sample; data are presented as median (B). Phage abundance in plasma and stool; data are presented as median (C). ∗∗ *p* < 0.01, ∗∗∗∗ *p* < 0.0001; plasma samples: HC *n* = 7, M0 *n* = 7, M3 *n* = 3, M6 *n* = 3; stool samples HC *n* = 2, M0 *n* = 5, M3 *n* = 2, and M6 *n* = 3.

Since viruses may be involved in the pathogenesis of MS, we sequenced the viruses of patients treated with ocrelizumab as the first-line therapy and compared it to virome of a healthy person from the same household. There are no significant differences between the newly diagnosed PwMS at M0 and their HC households at the level of alpha and beta diversity in plasma samples (Figure S4). However, several viral contigs show significantly different abundance between households and between the time points on ocrelizumab therapy (Figure 3A). We found that in post-treatment samples M3 and M6, the plasmatic viral pattern of PwMS is more similar to the HC from the same household than in the pre-treatment sample (M0).

We identified two groups of viruses, anelloviruses and bacteriophages, most prevalent and differentially abundant in the plasma of patients with MS and HC from the same households. We found that anelloviruses, non-pathogenic small ssDNA viruses, were the predominant eukaryotic viruses identified in the plasma virome. Their relative abundance was significantly higher in HC compared to newly diagnosed PwMS at M0 (*p* = 0.001), and their abundance non-significantly increased after treatment with ocrelizumab (Figure 3B), suggesting that short-term B cell depletion has some impact on these commensal viruses. In the most abundant group of phages, we observed significant differences during treatment (Figure 3C). Although, the changes were not as evident in the plasma samples, we observed a decline in phage abundance at M3, followed by an increase at M6. A similar but even more pronounced pattern was observed in the fecal samples. These alterations in the gut phage population, although limited by sample size, suggest a treatment-induced modulation of the human virome that may parallel or even precede the changes in gut bacteria. Although herpesviruses, including EBV, have been associated with the pathogenesis of MS, we did not detect herpesviral DNA in plasma or feces in our study cohort by either virome sequencing or targeted qPCR assays (data not shown).

### A persistent decrease in alpha diversity of the gut microbiota was only associated with those who had no response to ocrelizumab treatment

**Figure 4:**
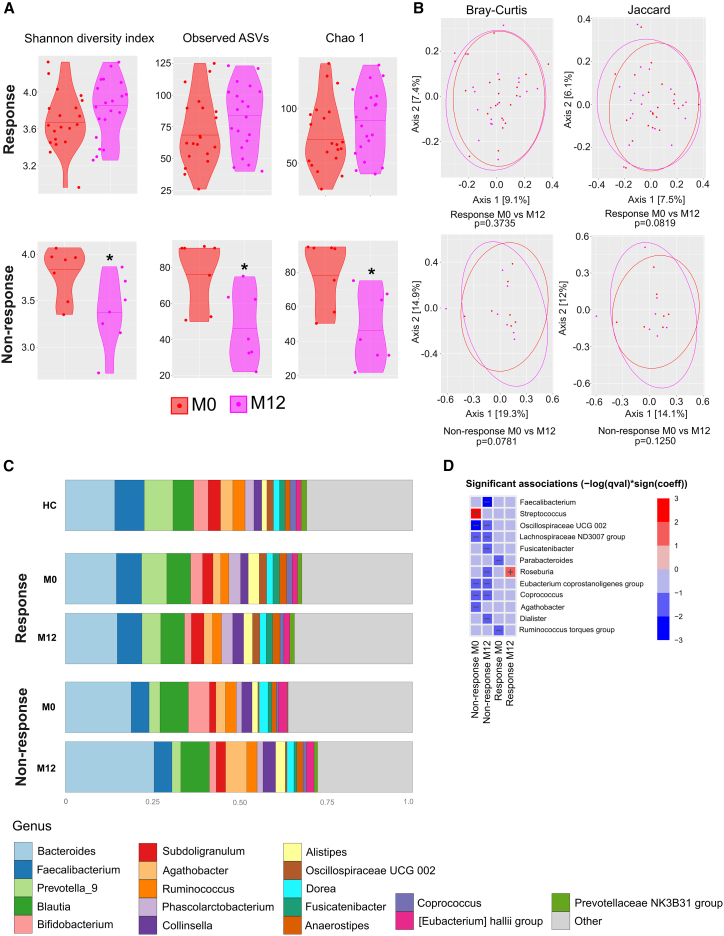
Pairwise analysis of the gut microbiota according to treatment response Alpha diversity is expressed as the Shannon diversity index, Observed ASVsf, and Chao1; data are presented as median (A). Beta diversity is expressed as the Bray-Curtis and Jaccard index (B). Abundances of the 18 most abundant bacterial taxa (C), and the heatmap showing significant associations of genera with specific group compared to HCs (D). ∗*p* < 0.05; HC: *n* = 81, response: *n* = 20, and non-response: *n* = 7.

To determine changes in the gut microbiota that were associated with treatment response outcomes, we compared the changes in the gut microbiota between M0 and M12 in PwMS who responded to ocrelizumab (*n* = 20) with those who did not (*n* = 7). While the alpha diversity of responders increased slightly, but not significantly, at the end of M12 compared to M0, the alpha diversity of non-responders decreased significantly (Figure 4A). There was no difference between responders and non-responders in alpha diversity at M0. After 12 months of therapy non-responders had significantly lower levels in alpha diversity as compared to responders (Figure S5). There were no significant shifts in beta diversity indices between M0 and the end of M12 in either group (Figure 4B).

Responders at both M0 and the end of M12 had more or less similar bacterial taxa as HCs, while non-responders differ in genera *Bacteroides*, *Bifidobacterium*, and *Agathobacter* (Figure 4C). Compared to HC, non-responders had significantly lower levels of genera *Agathobacter*, *Coprococcus*, *Eubacterium coprostanoligenes* group, *Lachnospiraceae* ND3007 group, and *Oscillospiraceae* UCG 002 and significantly higher levels of genera *Streptococcus* at M0. After 12 months (i.e., at the end of M12), they had significantly lower levels of genera *Dialister*, *Coprococcus*, *Eubacterium coprostanoligenes* group, *Roseburia*, *Fusicatenibacter*, *Lachnospiraceae* ND3007 group, *Oscillospiraceae* UCG 002, and *Faecalibacterium* compared to HCs. We did not detect any of these associations in treatment responders. Responders had significantly lower levels of *Parabacteroides* at M0, but this was not observed at the end of M12. In responders at the end of M12, only levels of genus *Roseburia* were higher compared to HCs (Figure 4D). All bacteria with significantly reduced abundance in non-responders are putative or recognized short-chain fatty acid (SCFA) producers.

### Treatment response was associated with augmentation of glycosphingolipid and steroid biosynthesis pathways that existed prior to the treatment

To assess the functional consequences of differences in the microbial community in treatment responders and non-responders, we used the PICRUSt2 tool to predict the functional potential of a bacterial community based on marker gene sequencing profiles. Here, we report only the significant differences between responders and non-responders assessed at two different times. Interestingly, we found significantly augmented pathways associated with cardiovascular diseases and cardiac muscle contraction in non-responders (Figure S6). Pathways associated with pancreatic secretion were also augmented in non-responders. Additionally, they had higher levels of endocytosis compared to responders at M0. Of great interest was the augmentation of the calcium signaling pathway in non-responders at M0. In responders, we found significant augmentation of glycosphingolipid and steroid biosynthesis.

After 12 months of treatment, we found that in responders, a significant augmentation of the steroid biosynthesis pathway was still present; we further noted that augmentation of glycosphingolipid biosynthesis appeared in non-responders (Figure S7). Moreover, responders had augmented pathways for other types of O-glycan biosynthesis as well as the retinoic acid-inducible gene 1 (RIG-1)-like receptor signaling pathway.

### Ocrelizumab treatment led to a decrease in total IgM after 12 months

The effect of ocrelizumab treatment targets CD20 antigens, which is a cell marker found principally on B cells. As such, the mechanism of action is considered to be mainly anti-inflammatory via selective depletion of B cells. Therefore, our next step was assessing the immunoglobulins and specific antibody levels in serum at M0, M6, and M12 and comparing it with HCs.

**Figure 5:**
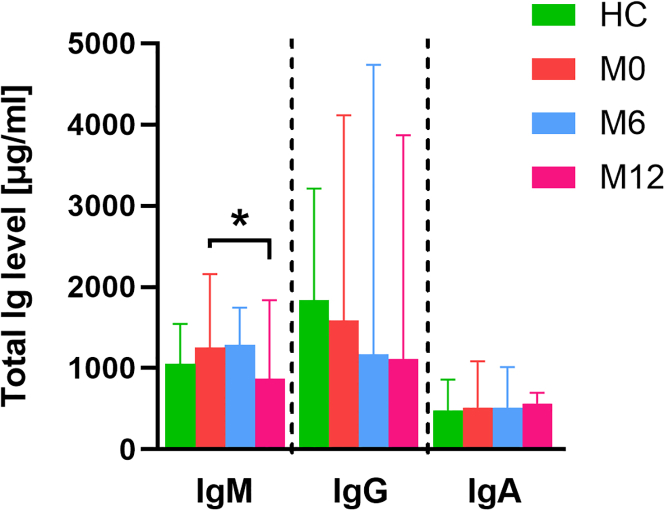
The level of total immunoglobulins in HC and PwMS over the course of treatment The Kruskal-Wallis test with Dunn’s multiple comparisons test was used to compare all time points to HCs, the Friedman test with Dunn’s multiple comparisons test was used to compare statistical differences between M0, and at the end of M6 and M12. Data are presented as median with 95% CI. ∗*p* < 0.05; HC *n* = 40 and MS *n* = 19.

First, we measured the levels of all three immunoglobulin isotypes: IgM, IgG, and IgA. During the course of treatment, there was a significant decrease in the level of IgM and only a decreasing trend in IgG levels (Figure 5). We did not observe any changes in IgA compared to HC or at M0.

### Ocrelizumab significantly changes levels of some anti-commensal antibodies

**Figure 6:**
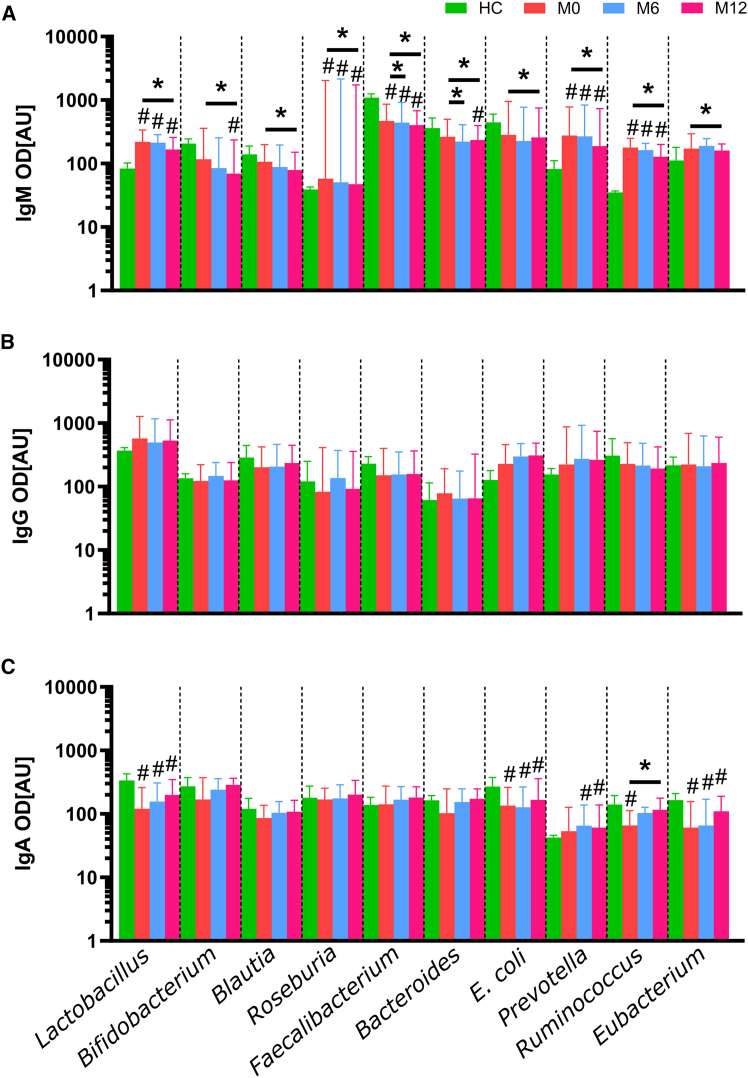
The level of anti-commensal antibodies in HCs and PwMS over the course of the treatment ∗Depicts significant differences in antibody levels compared to M0 (the Friedman test with Dunn’s multiple comparisons test), and # depicts significant differences compared to HCs (the Kruskal-Wallis test with Dunn’s multiple comparisons test). Data are presented as median with 95% CI. Green = HCs, red = M0, blue = M6, pink = M12. ∗#*p* < 0.05; HC *n* = 40 and PwMS *n* = 19.

The importance of gut barrier functions and gut microbiota to the pathogenesis of MS suggests that specific antibodies to gut commensal microbiota could be used as possible biomarkers. To see the effect of ocrelizumab on anti-commensal antibodies, we measured antibody levels against ten commensal bacteria that are a common part of the intestinal microbiota of people living in the Czech Republic (Figure 6).^15^^,^^16^

We found that PwMS had increased anti-commensal IgM antibodies against *Lactobacillus plantarum*, *Roseburia intestinalis*, *Prevotella ruminicola*, and *Ruminococcus flavefaciens* than HCs during whole 12 months of ocrelizumab treatment. In contrast, levels of IgM against *Faecalibacterium prausnitzii* were higher in PwMS compared to HC. All measured anti-commensal IgM antibodies decreased during ocrelizumab treatment similarly, as did the total IgM. There were no significant changes in IgG anti-commensal antibody levels. In IgA antibodies, PwMS had significantly lower levels of antibodies against *Lactobacillus plantarum*, *Escherichia coli*, *Ruminococcus flavefaciens*, and *Eubacterium rectale*. Interestingly, all analyzed IgA anti-commensal antibody levels increased slightly during treatment, with a significant increase in IgA against *Ruminococcus flavefaciens* at the end of M12 compared to M0. At the end of M12, PwMS had significantly higher IgA antibodies against *Prevotella ruminicola* than HCs (Figure 6).

### Changes in total immunoglobulin levels according to treatment response

**Figure 7:**
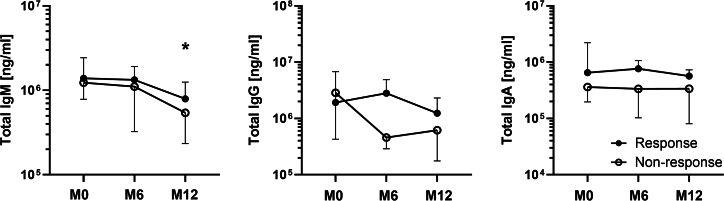
Levels of total IgM, IgG, and IgA according to the response to treatment ∗*p* < 0.05 depicts a significant decrease after 12 months of treatment in responders as compared to M0 tested using the Friedman test with Dunn’s multiple comparisons test; Data are presented as median with 95% CI. Responders: *n* = 12 and non-responders: *n* = 4.

Next, we performed the analysis of total immunoglobulins as well as specific anti-commensal antibodies against selected gut bacteria in PwMS according to the treatment response at M0 and at the end of M6 and M12 (Figure 7).

Responders had higher levels of IgM and IgA isotypes at M0 in comparison to non-responders. We found a significant decrease in IgM, but only in responders; however, the pattern of immunoglobulin levels in non-responders was similar. In non-responders, there was a steep decrease of total IgG from M0 to the end of M6 in non-responders (Figure 7).

Interestingly, we found a significant decrease in the level of specific IgM anti-commensal antibodies over the course of the treatment in responders (Figure S8). There were no significant changes in IgG in responders or non-responders (Figure S9); additionally, we found a significant increase in IgA antibodies in responders but only against *Ruminococcus flavefaciens* (Figure S10). Comparing these results with the abundance of those microbes in responders and non-responders, we found that the relative abundance of *Roseburia intestinalis* significantly decreased between M0 and the end of M12 in non-responders, resulting in a significant difference between non-responders and responders by the end of M12. In contrast, the abundance of *R*. *intestinalis* in responders increased after 12 months of treatment. Next, we found that non-responders had higher levels of all antibody isotypes against *F. prausnitzii*. At the end of M12, non-responders had decreased levels of antibodies against *F*. *prausnitzii*; however, this difference was not statistically significant. While we observed a significant decrease in the relative abundance of *Bifidobacterium adolescentis* in non-responders at the end of M12, we observed only increased IgA against *B*. *adolescentis* at the end of M12 in this study group.

### Elevated levels of molecules associated with microbial translocation in multiple sclerosis

Realizing that a disruption of the gut barrier can be followed by an inflammatory response provides insights into a better understanding of the mechanisms by which changes in intestinal permeability contribute to neuroinflammation. Therefore, we analyzed serum molecules that we previously described as important molecules related to gut barrier damage and the pathogenesis of inflammatory bowel diseases and anti-TNF-α treatment.^15^

**Figure 8:**
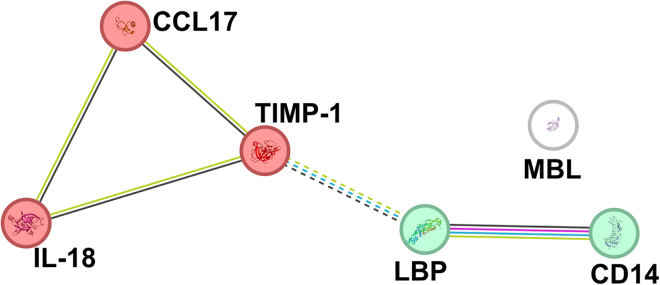
Protein-protein interaction network functional enrichment analysis The network was produced using the STRING database and STRING consortium 2023 web source. The interconnection biomarkers shown by protein-protein network functional enrichment analysis are based on co-expression (black line), experimentally determined (pink line), and both text mining (yellow line), and curated databases (blue line). K-means clustering revealed two distinct clusters (shown by the color of the nodes), and inter-cluster edges are represented by dashed lines.

Next, we utilized protein-protein interaction network functional enrichment analysis accompanied by K-mean clustering to gain insight into the interaction between these molecules (Figure 8). Two major clusters were identified, suggesting a link between (1) molecules involved in intestinal barrier damage (lipopolysaccharide-binding protein [LBP], soluble CD14 [CD14], and mannose-binding lectin [MBL]) and (2) the subsequent immune response against the microbiota, and its regulation (tissue inhibitor of metalloproteinase-1 [TIMP-1], interleukin-18 [IL-18], C-C motif chemokine ligand 17 [CCL17]).

**Figure 9:**
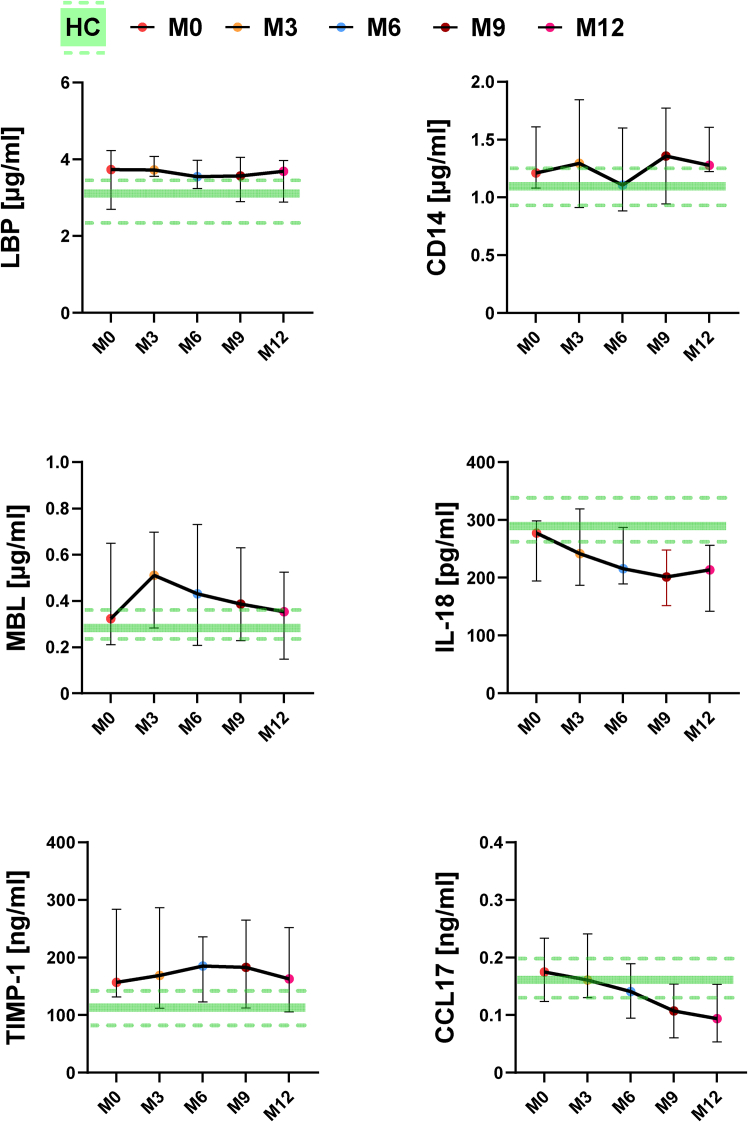
Levels of serum biomarkers in newly diagnosed PwMS undergoing ocrelizumab treatment Lipopolysaccharide-binding protein (LBP), soluble CD14 (CD14), Mannose-binding lectin (MBL), Tissue inhibitor of metalloproteinase-1 (TIMP-1), Interleukin-18 (IL-18), C-C motif chemokine ligand 17 (CCL17). Data are presented as median with 95% CI. Green lines depict the median and 95% CI of health controls (HCs). (HC *n* = 61, M0 *n* = 25, M3 *n* = 23, M6 *n* = 25, M9 *n* = 13, and M12 *n* = 16).

First, we analyzed levels in PwMS without prior therapy treated with ocrelizumab, and second, we coupled these results with an analysis of those PwMS with previous interferon beta treatment failure. Compared to HC, the newly diagnosed PwMS without prior therapy had slightly elevated but not significant levels of LBP, CD14, MBL and TIMP-1 during treatment, while there was a slight decrease in levels of CCL17 (Figure 9). This may indicate stimulation of the immune system by microbial components such as lipopolysaccharide (LPS) and mannose, which have the potential to cause low-grade inflammation.^17^ These differences in the level of LBP, CD14 at M0, and the end of M3 and M6 were significant in PwMS with previous interferon beta treatment failure (Figure S11). In newly diagnosed PwMS patients undergoing ocrelizumab treatment, we observed a continuous decrease in IL-18 and CCL17 compared to HCs (Figure 9). This decreasing pattern was not observed in the PwMS previous interferon beta treatment failure. Compared to HCs, the level of CCL17 began to increase by the end of month six and continued to increase until the end of month 15 (Figure S11).

**Figure 10:**
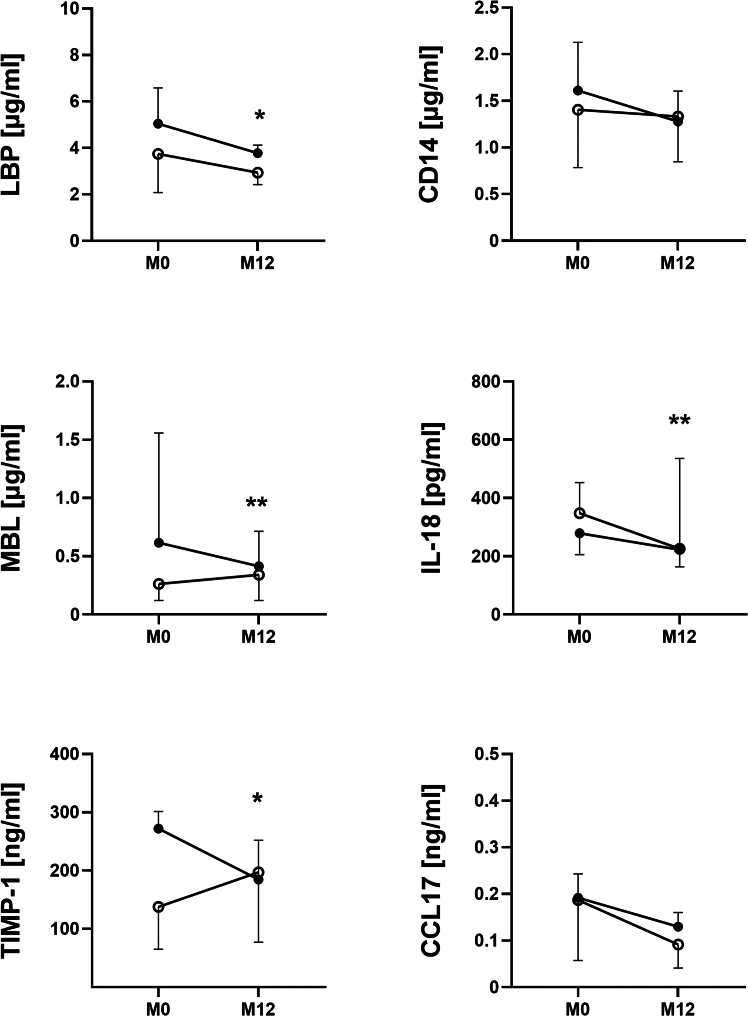
Levels of serum biomarkers in PwMS according to treatment response Lipopolysaccharide-binding protein (LBP), soluble CD14 (CD14), mannose-binding lectin (MBL), tissue inhibitor of metalloproteinase-1 (TIMP-1), interleukin-18 (IL-18), C-C motif chemokine ligand 17 (CCL17). ∗*p* < 0.05 or ∗∗*p* < 0.01 depicts a significant decrease after 12 months of treatment in responders. Data are presented as median with 95% CI. Responders: *n* = 17 and non-responders: *n* = 7.

Next, we compared median levels of LBP, CD14, MBL, IL-18, CCL-17, and TIMP-1 between responders and non-responders before and after 12 months after ocrelizumab treatment. After 12 months of treatment, there was a significant decrease in LBP, MBL, TIMP-1, and IL-18, but only in responders. There were no significant differences between responders and non-responders at M0 and M12 (Figure 10).

## Discussion

Studies in animal models of MS and observations in PwMS have suggested a link between the microbiota and the pathogenesis, course, and progression of MS as well as functional changes in response to treatment.^18^ One of the main effects of the gut microbiota is its influence on the integrity of the intestinal barrier. Disruption of this barrier and the subsequent translocation of gut microbes and their components lead to low-grade systemic inflammation. To date, no specific microbial signature has been found in PwMS across different populations. Although reduced alpha diversity has been found in most studies, there is no consensus on what specific changes occur in the gut microbiota.^2^^,^^19^^,^^20^ Disease-modifying treatments alter the abundance of some gut bacteria,^21^ and some gut bacteria appear to contribute to MS treatment effectiveness.^14^ Therefore, we analyzed the gut microbiota of PwMS, followed changes during ocrelizumab treatment, and investigated how ocrelizumab’s B cell-depleting treatment affected antimicrobial antibody responses and markers of gut barrier integrity.

We found that PwMS had a different gut microbiota composition than HCs, although the differences often depended on the duration of ocrelizumab treatment. To examine the effect of duration, we compared the gut microbiota of newly diagnosed treatment-naïve PwMS (clinically isolated syndrome - CIS cohort) with that of HCs. Alpha diversity between newly diagnosed PwMS before ocrelizumab treatment and HCs was not statistically different, although newly diagnosed PwMS tended to have lower diversity. A similar pattern has been reported by other studies, suggesting that the gut microbiota either takes longer to change after MS onset or is mainly influenced by MS treatment.^22^ For this reason, we would like to emphasize the importance of longitudinal sampling performed in our study. Compared to HCs, newly diagnosed PwMS have significantly higher abundances of *Barnesiella* spp. and *Streptococcus* spp. and significantly lower abundances of *Prevotella_9*, *Coprococcus* spp., *Agathobacter* spp., and the *Oscillospiraceae* NK4A214 group. This is in agreement with other studies on CNS damage where a similar pattern was observed,^23^ e.g., decreased *Coprococcus* spp. is associated with various neurological disorders and *Agathobacter* spp. attenuate microglia-mediated neuroinflammation in Alzheimer’s disease. Interestingly, the latter two microbes can dampen inflammation via their production of butyrate.^24^^,^^25^

Unlike cross-sectional studies, we were able to track changes in the microbiota during treatment in each individual. We found that the alpha diversity of gut bacteria in newly diagnosed PwMS transiently decreases during the first year of ocrelizumab treatment, with a loss of diversity occurring after 6 months. Even though this is not explicitly discussed in the literature, a similar transient decrease was reported in a previously published study,^22^ suggesting it is a common feature of ocrelizumab treatment. We found that during this decrease, there was a significant decrease in *Blautia* spp., which was associated with MS and the risk of relapse.^26^^,^^27^^,^^28^ While the alpha diversity of PwMS at the end of M12 resembles that of controls, they had significantly more *Collinsella* spp. in their stool as compared to M0. Contrary to our finding there was reported no changes in abundance of *Collinsella* spp. in PwMS compared to HC.^29^

While there were no significant changes in beta diversity indices during ocrelizumab treatment, the abundance of some microbes differed from HCs at specific times, while other microbes differed in abundance most of the time, suggesting permanent changes. These latter changes were characterized by a lower abundance of *Faecalibacterium* spp. and a higher abundance of *Barnesiella* spp. Low abundance of *Faecalibacterium* spp. in MS has been previously reported.^30^^,^^31^^,^^32^ *F. prausnitzii* is a dominant member of the genus *Faecalibacterium* in the feces of healthy humans.^33^ It reduces inflammation by promoting regulatory T cell functions and improves intestinal barrier function, both of which could directly counteract the MS pathogenesis.^19^^,^^34^^,^^35^^,^^36^ The increase in *Barnesiella* spp. in PwMS is not consistent with its proposed effect on the immune system. It could also decrease inflammatory tuning of the host’s immune system by protecting the gut mucosa from pathogenic bacteria and by driving the development of several immunoregulatory cells, which have been shown to increase resistance to arthritis in animal models.^37^ We observed a transiently decreased abundance of genera *Oscillospiraceae* UCG 002, *Agathobacter*, and a higher abundance of *Streptococcus* at baseline (M0). The *Oscillospiraceae* family can degrade dietary fiber into the anti-inflammatory butyrate; additionally, we found an increase in the genus *Alistipes* at the end of M12 and *Phascolarctobacterium* at the end of M9, both of which are producers of SCFA with putative anti-inflammatory properties.^38^^,^^39^ These results provided an unique insight into the microbial profile during ocrelizumab treatment, with the profile mainly showing changes in SCFA producers. SCFA, such as acetate, propionate, and butyrate, are important metabolites in the maintenance of the intestinal barrier, host metabolism, immune tolerance, and immunity functions.^40^ Reduced production of SCFA is associated with a leaky gut, dysregulation of antigen presentation, and activation of Th1, Th17, Tc, and B cells. This could lead to microbial translocation, increased systemic inflammation, and even promote some MS comorbidities such as metabolic syndrome.^41^

Interestingly, we found a similar decrease in alpha diversity in PwMS who had previously relapsed on IFN-β treatment. Compared to newly diagnosed PwMS, this decrease was slightly delayed from six months to 9–12 months of ocrelizumab treatment, and while the alpha diversity increased slightly by the end of M15, it never reached HC-like levels. This difference from the newly diagnosed PwMS cohort suggests that dysbiosis is affected by both the disease’s duration and previous treatment. This is supported by our finding that the abundance of genus *Prevotella_9* was increased at M0 and at the end of M3, and only then did it start to decrease to HC levels. Moreover, recent study by Gupta et al. 2025 showed that new-onset, untreated PwMS show significant reductions in IgA-bound fecal microbiota, as well as changes in the abundance and prevalence of specific gut bacterial strains. Further, they identified specific organisms where IgA-coating patterns were lost following B cell-depleting therapy, as well as organisms whose IgA-coating patterns shifted to align more closely with controls.^42^ We hypothesize that there are at least two simultaneous mechanisms that could influence the alpha diversity of the microbiota under treatment with ocrelizumab. The first mechanism is the direct effect of the first dose of ocrelizumab (the complete and rapid depletion of B cells) and the second mechanism is the overall consequence of the reduction of the proinflammatory state (improvement of MS) or non-reduction (no response to ocrelizumab treatment) leading to the restoration or non-restoration of the gut microbiota from dysbiosis.

Beta diversity was significantly different from HC at all time points except at the end of M15, suggesting that ocrelizumab treatment may be able to normalize the gut microbiota in PwMS who had previously relapsed on IFN-β. Three months after starting treatment, we found a significantly lower abundance of genera *Ruminococcus*, *Christensenellaceae* R7 group, and *Coprococcus* compared to HC. Both taxa, *Ruminococcaceae* NK4A214 and *Christensenellaceae* R7 group have been identified as members of the microbiota that contribute to human health by producing SCFA and having anti-inflammatory effects; both are consistently decreased in pediatric-onset PwMS.^43^ After 12 months of treatment in PwMS who had previously relapsed on IFN-β, we found a significant decrease in genera *Dorea* and *Ruminococcus torques* group, which are thought to be major degraders of intestinal mucin glycoprotein^44^ and thus could be directly involved in making gut epithelium accessible to intestinal bacteria in PwMS.

Next, we hypothesized that the gut microbiota could predict the therapeutic success of ocrelizumab treatment and compared the composition of the gut microbiota of PwMS who responded to ocrelizumab to those who did not respond. We found that the alpha diversity of the gut microbiota decreased significantly during 12 months of ocrelizumab treatment in non-responders vs. a non-significant increase trend in responders. This suggests that the reduced diversity often described in inflammatory diseases may be its consequence and not the cause. Although our data support this provocative hypothesis, a different study is needed to demonstrate it decisively. There were no significant differences in beta diversity between responders and non-responders, although some microbes were associated with specific states. Compared to HCs, non-responders had significantly decreased *Oscillospiraceae* UCG 002, *Lachnospiraceae* ND3007 group, *Eubacterium coprostanoligenes* group, and *Coprococcus* spp. in both time points and markedly decreased *Faecalibacterium* spp. at the end of M12. This suggests that non-responders had a sustained reduction in the level of bacteria involved in SCFA production and gut barrier maintenance. Interestingly, a similar pattern of reduction in recognized or putative SCFA producers was found in another neurological disorder - Parkinson’s disease.^45^ The similarities in the gut microbiota between MS and Parkinson’s disease could suggest some unexplored pathogenetic mechanisms common to both. The *Eubacterium coprostanoligenes* group promotes mucin secretion by goblet cells, thereby strengthening the integrity of the intestinal mucus barrier, and reducing microbial invasion and subsequent inflammatory responses.^46^ A previous study reported a higher abundance of *F. prausnitzii* in PwMS treated with anti-CD20 compared to untreated patients.^20^ However, we found not only a decrease in its abundance during ocrelizumab treatment, but that this decrease was significant only in non-responders.

The responders were generally more similar to HCs and differed only in their decreased abundances of genera *Parabacteroides* and *Ruminococcus torques* group at M0 and an increased abundance of *Roseburia* spp. at the end of M12. *Roseburia* spp. reduces the inflammatory tuning of the immune system and alleviates neuroinflammation by producing SCFA. This could reduce microglia activation, prevent the production of pro-inflammatory cytokines in the brain, and promote the maintenance of the intestinal barrier.^47^^,^^48^^,^^49^ Successful treatment with ocrelizumab normalized the abundance of *Parabacteroides* spp., which was initially decreased compared to HCs (at M0, before treatment). This may reduce neuroinflammation; we recently described that oral administration of *Parabacteroides distasonis* antigens prevent severe forms of EAE by modulating T cell priming.^50^ These results suggest that responders are differentiated from non-responders by gut microbes that prime the immune system toward an anti-inflammatory state and improve maintenance of the gut barrier. These microbes could induce Treg cell maturation, thus reverting the Treg/Th17 cell imbalance in MS and even making their hosts more resilient to neuroinflammation.^51^ These mechanisms could synergize with ocrelizumab and improve its therapeutic effect.

Next, we predicted the metabolic pathways of the gut microbiota and linked them to the response to ocrelizumab. We found that the responder’s microbiota already had significantly enriched metabolic pathways for glycosphingolipid and steroid biosynthesis at M0. Glycolipids are major components of CNS myelin, suggesting that enrichment of the glycosphingolipid synthesis pathway may, in turn, lead to the promotion of oligodendrocyte differentiation and increased myelin sheath repair, thus contributing to the overall treatment response.^52^ Considering that steroids can be used as drugs to treat MS relapses, the enrichment of the steroid biosynthesis pathway in responders could contribute to the reduction of inflammation in responders as early as M0. Enrichment of steroid biosynthesis pathways remained elevated in responders even after 12 months of ocrelizumab treatment. In non-responder’s microbiota, cardiovascular disease-related pathways were enriched at M0, which suggests the link between gut microbiota and increased cardiovascular risk in PwMS, described in the current literature.^53^^,^^54^ The calcium signaling pathway was another significantly enriched pathway in non-responders at M0. Disruption of Ca^2+^ homeostasis could drive various neurodegenerative and neuroinflammatory pathways by disturbing the function of mitochondria, lysosomes, and endoplasmic reticulum. The Na^+^-Ca^2+^ exchanger regulates Ca^2+^ homeostasis in various cell types, including neurons, astrocytes, and microglia. Alterations in its activity have been linked with neurodegenerative processes in various neurodegenerative models like Parkinson’s disease.^55^ Thus, intracellular Na^+^ and Ca^2+^ imbalance drives neuro-axonal dysfunctions, failure of myelin repair mechanisms, and activation of antigen-independent T-cells.^56^^,^^57^^,^^58^^,^^59^ After 12 months of treatment, non-responders had significant enrichment of both the bacterial invasion of the epithelial pathway and the RIG-1-like receptor signaling pathway. This was surprising because PwMS suffer from insufficient expression of MDA5 and RIG-1 as compared to HC.^60^ RIG-1-like receptors are a type of intracellular pattern recognition receptors involved in the recognition of viruses by the innate immune system and triggering antiviral interferon responses. This suggests that bacterial as well as viral dysbiosis in gut microbiota in non-responders is associated with gut barrier dysfunction, as the RIG-1/MAD5/MAVS signaling promotes gut integrity via protective interferon response in the intestinal epithelium.^61^

Next, we analyzed systemic exposure to viruses, which is rarely studied in PwMS.^62^^,^^63^ Although there were no differences in alpha or beta diversity, we identified several contigs with different abundance between PwMS and HC. PwMS have significantly lower abundance of family *Anelloviridae*, which non-significantly increases after ocrelizumab treatment. This suggests that the altered anellovirus dynamics may reflect the immunological status of the host, which is related to the presence of anelloviruses^64^ in PwMS even prior to treatment. Furthermore, during ocrelizumab treatment, we observed a marked decrease in bacteriophage abundance in plasma and stool samples, which mirrored similar changes in bacterial diversity. While these data represent only limited cohort, they suggest that early shifts in phage populations could contribute to, or even drive, later bacterial changes, impacting host immune interactions.^65^^,^^66^ Although herpesviruses, including EBV, have been associated with the pathogenesis of MS, we were unable to detect herpesviral DNA in plasma or feces in our study cohort by either virome sequencing or targeted qPCR assays (data not shown). This could be due to the fact that there was no active infection.^67^ Further data on changes in the viral composition of PwMS during anti-CD20 treatment are essential for understanding its overall role in the disease manifestation and progression and its side effects.

Mucosal (intestinal) immunity plays an important role in shaping the microbiota in health as well as in neuroimmune diseases. It is involved in the bidirectional communication called microbiota-gut-brain axis. This communication is composed from neural, hormonal and immune pathways.^68^^,^^69^^,^^70^^,^^71^ B cells play an important role in the pathogenesis of MS and B cell-targeted treatments are used in MS. Therefore, we compared serum immunoglobulins in HCs and PwMS receiving ocrelizumab. While we found no significant difference between the HC and clinically isolated syndrome (CIS) cohort, we found a decrease in total IgM after 12 months of ocrelizumab treatment. There were no significant differences in the other isotypes over time, although IgG showed some tendency to decrease. The reason for this finding is that anti-CD20 treatment does not influence the long-lived plasma cells, which lack CD20 so IgM antibody levels often remain stable, at least initially.^72^^,^^73^ Some studies have reported a gradual decline in IgM levels. However, these changes are more pronounced over a longer period of time and may not be evident in the first six to 12 months of treatment, as reviewed by Saidha et al. 2023.^74^ Unlike IgG, IgM antibodies are associated with axonal damage in MS,^75^ suggesting that this result reflects the biological effect of ocrelizumab on plasma cells.

Next, we found that PwMS have altered levels of antibodies against the ten most common gut commensal bacteria and that this profile is changed by ocrelizumab treatment. This was unexpected since ocrelizumab treatment should have no effect on the plasma cells in the intestine.^76^ All these changes were again particularly strong for the IgM and in some IgA, but not in IgG. We found that PwMS at M0 have more IgM antibodies to *Lactobacillus plantarum*, *Roseburia intestinalis*, *Prevotella ruminicola*, and *Ruminococcus flavefaciens* and less to *F. prausnitzii*. This general increase in IgM levels suggests that higher antibody levels may be induced by changes in microbiota composition as well as by translocating microbes due to an impaired intestinal barrier in PwMS that stimulates intestinal B cells. But as we demonstrated with *F*. *prausnitzii*, it is not that straightforward. Previously, it was thought that *Faecalibacterium* spp. influenced the immune system mainly through the production of SCFA,^77^ but it was later discovered that it produces a microbial anti-inflammatory molecule (MAM) that can significantly attenuate inflammation regardless of the number of bacteria.^78^ Moreover, MAM is not the only bacterial effector that mediates the positive effects of *Faecalibacterium* spp.^79^ Thus, the immune response to this bacterium may actually decrease when gut permeability increases. When we separated the cohort into ocrelizumab responders and non-responders, the non-responders had higher levels of antibodies against *Faecalibacterium* spp. than responders; we additionally found a significant decrease in IgM at the end of M12. Intestinal barrier damage and dysbiosis often occur together since they are both associated with a vicious circle of inflammation and damage. To this end, we selected markers related to the response to LPS. PwMS have low-grade endotoxemia, likely due to an impaired intestinal barrier function.^80^^,^^81^^,^^82^ LPS is known to increase blood-brain barrier permeability and exert pro-inflammatory effects on microglia and astrocytes.^83^ Therefore, we analyzed serum biomarkers of gut barrier dysfunction and inflammation during ocrelizumab treatment and between ocrelizumab responders and non-responders. We found that these cytokines are not significantly different in newly diagnosed PwMS receiving ocrelizumab compared to HCs, but there are significant differences in PwMS treated after IFN-β treatment failure, i.e., they have increased levels of LBP and soluble CD14 in the first six months of ocrelizumab treatment before they normalize between M9–M15. Previous work found increased levels of LPS, LBP, and CD14 in PwMS,^81^^,^^82^^,^^84^ although our results showed that this increase was not found in newly diagnosed PwMS or effectively treated PwMS. This suggests more severe gut barrier damage and microbial translocation in these PwMS and may even explain why previous treatment was unsuccessful. Similar to the microbiota composition, these factors also showed different dynamics between responders and non-responders during 12 months of ocrelizumab treatment. While there were no significant differences between responders and non-responders, LBP and MBL decreased significantly during treatment, but only in responders, while there were no changes in non-responders. This suggests that the response of PwMS to the low-grade inflammation caused by the release of microbial components into the bloodstream may be related to the success of the ocrelizumab treatment. These results demonstrate a potential link between gut barrier damage and the subsequent immune response associated with microbial translocation and MS pathogenesis and treatment. They also highlight the important differences between ocrelizumab-treated newly diagnosed PwMS and PwMS who failed IFN-β treatment. This information can be the basis for further studies as well as the identification of PwMS who will likely benefit from ocrelizumab treatment; however, further studies are needed to identify the potential use of these molecules to personalize MS treatment.

### Limitations of the study

This study has several limitations. First, we were not able to analyze all serum samples from all responders and non-responders for whom we performed microbial profiling due to small sample volumes in some cases. Despite this limitation, we were able to perform all these analyses at M0 and M12 in a paired manner and compared data from M0 and the end of M12. Another limitation of our study is the restricted availability of biological material, which prevented us from performing a more comprehensive virome analysis in stool and plasma. However, the inclusion of household controls provides an important comparative framework that strengthens the interpretation of our results despite this constraint. IgM levels decreased significantly, but only in responders, whereas in non-responders, we observed only a similar trend. Since there were no significant differences in IgM levels between responders and non-responders at the end of M12, IgM probably does not reflect the therapeutic efficacy of the treatment. However, this may have been limited by the number of non-responders in this analysis. Nevertheless, all these analyses were paired. Second, although we predicted functional capacities with PICRUSt2, these results warrant proof of this prediction by metabolomics; consequently, a key next step will be to perform targeted or untargeted metabolomic analyses on the same samples to validate these predictions. Third, we were not able to support our immune molecules analyses in serum with the measurement of inflammatory molecules in cerebrospinal fluid and thus we were not able to show a link between immune markers in the circulation and those in the cerebrospinal fluid; this would have been valuable information to help us better understand the inflammatory response in PwMS during treatment with ocrelizumab.

### Conclusion

In this single-center study, we demonstrated changes in gut microbiota, the gut barrier, and inflammatory markers, as well as antibody profiles during ocrelizumab treatment in newly diagnosed, treatment-naïve PwMS as well as in PwMS with previous relapse during IFN-β treatment. We also showed differences in PWMS depending on their response to ocrelizumab. We found an existing slight shift in the alpha diversity of the gut microbiota in CIS PwMS. During 12 months of treatment, we observed that the alpha diversity significantly but transiently decreased after six months of treatment. While an increasing trend towards to HCs was observed in responders at the end of month 12, non-responders continued to have low alpha diversity at this time point. These changes were related to the lower abundance of SCFA producers. Higher levels of biomarkers associated with intestinal barrier damage and inflammation, as well as the shifts in anti-microbial antibody levels we found, imply intestinal barrier damage in PwMS, with only responders showing a significant decrease in LBP and MBL after 12 months of treatment, suggesting a reduction in low-grade inflammation and gut barrier damage associated with a response to treatment.

## Resource availability

### Lead contact

Requests for further information should be directed to and will be fulfilled by the lead contact, Zuzana Jiraskova Zakostelska, (zakostelska@biomed.cas.cz).

### Materials availability

This study did not generate new materials or new unique reagents.

### Data and code availability

• De-identified individual patient-level data reported in this paper will be shared by the lead contact upon request.
• This paper does not report the original code.
• Any additional information required to reanalyze the data reported in this paper is available from the lead contact upon request.
• Repository NCBI bioproject: PRJNA1194976 and ENA: PRJEB83221.

## Acknowledgments

The authors thank Alena Kubatova for excellent technical assistance and all nurses from General Medical Hospital in Prague who assisted us with the study and Martin Mihula for sample preparation. We acknowledge the CF Genomics of 10.13039/501100006435CEITEC supported by the NCMG research infrastructure (LM2023067 funded by MEYS CR) for their support in obtaining the scientific data presented in this paper.

This research was supported by grants from the 10.13039/100009647Ministry of Health of the Czech Republic (NU20-04-00077), the Ministry of Education, Youth and Sports of the Czech Republic grant Talking microbes-understanding microbial interactions within One Health framework (CZ.02.01.01/00/22_008/0004597), the National Institute of Virology and Bacteriology Project (EXCELES Program, LX22NPO5103), funded by the 10.13039/501100000780European Union, 10.13039/100031478Next Generation EU, and the Academy of Sciences of the Czech Republic (LQ200202105).

## Author contributions

H.T.-H., E.K.H., M.K., Z.J.Z., S.C., and R.T. conceived and designed the research. S.C., Z.J.Z., and M.K. wrote the manuscript. V.T., M.P., P.K., J.L.P., I.K., and E.K.H. examined the patients and healthy controls and collected samples. Z.J.Z., T.T., T.H., S.C., R.R., M.S., and D.K. conducted the experiments and analyzed the data. S.C., J.K., M.S., and D.K. performed and interpreted the bioinformatic analysis and sequencing data. All authors critically revised the manuscript for important intellectual content and approved the final manuscript.

## Declaration of interests

The authors declare no competing interests.

## STAR★Methods

### Key resources table

**Table:** 

REAGENT or RESOURCE	SOURCE	IDENTIFIER
**Antibodies**		

Peroxidase-conjugated AffinniPure F(ab’)2 fragment goat anti-human Fc fragment specific (IgG)	Jackson ImmunoResearch Laboratories	Cat#109-036-170; RRID: AB_2783740
Peroxidase-conjugated AffinniPure F(ab’)2 fragment goat anti-human Fc fragment specific (IgA)	Jackson ImmunoResearch Laboratories	Cat#109-036-011; RRID: AB_2337592
Peroxidase-conjugated AffinniPure F(ab’)2 fragment goat anti-human Fc fragment specific (IgM)	Jackson ImmunoResearch Laboratories	Cat#109-036-129; RRID: AB_2337598

**Bacterial and virus strains**		

*Lactobacillus plantarum*	Institute of Animal Physiology and Genetics of the CAS	–
*Bifidobacterium adolescentis*	Institute of Animal Physiology and Genetics of the CAS	–
*Blautia coccoides*	Institute of Animal Physiology and Genetics of the CAS	–
*Roseburia intestinalis*	Institute of Animal Physiology and Genetics of the CAS	–
*Eubacterium rectale*	Institute of Animal Physiology and Genetics of the CAS	–
*Faecalibacterium prausnitzii*	Institute of Animal Physiology and Genetics of the CAS	–
*Ruminococcus flavefaciens*	Institute of Animal Physiology and Genetics of the CAS	–
*Bacteroides thetaiotaomicron*	Institute of Animal Physiology and Genetics of the CAS	–
*Prevotella ruminicola*	Institute of Animal Physiology and Genetics of the CAS	–
*Escherichia coli*	Institute of Animal Physiology and Genetics of the CAS	–

**Biological samples**		

serum	this study	–
stool	this study	–

**Chemicals, peptides, and recombinant proteins**		

CCL17/TARC	R&D systems	Cat#DY364
Interleukin-18	R&D systems	Cat#DY318-05
Mannose-Binding Lectin	R&D systems	Cat#DY2307
Lipopolysaccharide-Binding Protein	R&D systems	Cat#DY870
Soluble CD14	R&D systems	Cat#DY383
Tissue Inhibitor of Metalloproteinase 1	R&D systems	Cat#DY970
ZymoBIOMICS DNA Miniprep Kit	Zymo Research	Cat#D4300
KAPA HiFi HotStart Ready Mix	Roche	Cat#07958927001
QIAxcel DNA Screening Kit (2400)	QIAgen	Cat#929004
SequalPrep™ Normalization Plate Kit	Thermo Fisher Scientific	Cat#A1051001
DNA Clean & Concentrator Kit	Zymo Research	Cat#D4034
KAPA HyperPlus Kit	Roche	Cat#KK8503/07962355001
QIAamp viral RNA mini kit	Qiagen	Cat#52904
WTA 2 kit	Sigma-Aldrich	Cat#41121800
Nextera XT kit	Illumina	Cat#15032354
Tween® 20	Merck	Cat#P1379-500 ML
Bovine Serum Albumin	Merck	Cat#A7030

**Deposited data**		

NCBI	this study	PRJNA1194976
ENA	this study	PRJEB83221

**Oligonucleotides**		

341F GTCCTACGGGNGGCWGCAG	Generi Biotech	custom made
806R GGACTACHVGGGTWTCTAAT	Generi Biotech	custom made

**Software and algorithms**		

R statistics platform ver 4.3	R Foundation for Statistical Computing	https://www.r-project.org/
GraphPad Prism statistical software 8.1.1	GraphPad Software	https://www.graphpad.com/scientific-software/prism/
BioRender	BioRender website	https://www.biorender.com/

### Experimental model and study participant details

#### Study cohorts

Persons with multiple sclerosis were recruited between November 2018 to December 2020 from the Center for Demyelinating Diseases, Dpt. of Neurology, General University Hospital, and Charles University, Prague, 1^st^ Faculty of Medicine. The studied cohort included 25 newly diagnosed PwMS, which were treatment-naïve and recruited from new admissions to the MS clinic (with clinically isolated syndrome, CIS cohort) and 9 PwMS on IFN-β who had relapsed and were indicated for treatment with ocrelizumab (IFN-β cohort) (**Demographic table**). The eighty-one healthy control (HC) subjects were also recruited at the Center for Demyelinating Diseases, Dpt. of Neurology, General University Hospital, and Charles University, Prague, 1^st^ Faculty of Medicine in the Czech Republic. Inclusion criteria for PwMS and HCs were white ethnicity and age 18–60 years. The exclusion criteria for PwMS and HCs were gastrointestinal, psychiatric, or mental disorders and the use of antibiotics in the three months prior to sampling. Age-matched and sex-matched HCs were recruited as healthy controls (**Demographic table**).

#### Ethics approval and consent to participate

All study participants signed an informed consent. This study was approved by the Ethics Committee of General University Hospital, Prague (15/19 Grant AZV VES 2020 VFN; 23.5.2019).

#### Examination

All PwMS were examined during their recruitment by the same neurologist. The collected data included age, sex, disease duration, expanded disability status scale (EDSS) assessments, and MS functional composite (MSFC) assessments. All study participants underwent a baseline cerebral MRI scan and yearly follow-up scans to evaluate radiological disease activity based on the number of new expanding T2 brain lesions.^85^ A relapse was defined according to the 2017 McDonald diagnostic criteria.^86^ The number of relapses, a worsened EDSS, and new expanding white matter lesions on MRI were used for calculation of NEDA-3 at baseline and after 12 months (i.e., no evidence of disease activity, i.e., no relapses, no new/enlarging white matter MRI lesions, and stable EDSS). We define NEDA-3 in our study cohort as really no evidence of disease activity, therefore even one new lesion on MRI is excluding PwMS as being NEDA-3. PwMS without a NEDA-3 were judged as non-responders to treatment retrospectively.^85^ Stool and fasting blood samples from newly diagnosed, treatment naive PwMS and PwMS who were indicated for treatment with ocrelizumab after relapse on IFN-β were collected before the beginning of treatment and every 3 months for a minimum of 12 months.^15^^,^^87^ All samples were aliquoted and stored at −80 °C until processing.

#### Evaluation of disease severity and treatment response

**Table:** Demographic table: Summary of anthropometric and clinical parameters at baseline (prior treatment to ocrelizumab) and study endpoint (1 year)

	Sex (F/M)	Age	BMI	EDSS at baseline	EDSS at endpoint	Age of disease onset	Disease duration
Treatment naïve PwMS (*n* = 25)	18/7	40.0 (31.5, 50.5)	24.5 (22.0, 30.1)	3.0 (2.0, 4.0)	2.5 (2.0, 4.0)	38.0 (29.5, 48.0)	2.0 (2.0, 3.0)
PwMS with relapse under IFN-β treatment (*n* = 9)	6/3	46.0 (34.5, 48.5)	25.7 (20.8, 33.0)	2.0 (1.5, 2.5)	1.8 (1.5, 2.1)	34.0 (26.0, 41.5)	13.0 (5.0, 16.0)
Healthy controls (*n* = 81)	42/39	39.0 (27.5, 44.5)	24.4 (21.6, 28.8)	–	–	–	–
	*p* = 0.1059 (CIS vs. HC) *p* = 0.4943 (IFN- β vs. HC) *p* > 0.9999 (CIS vs. IFN- β	*p* = 0.1188	*p* = 0.6876	*p* = 0.014	*p* = 0.057	*p* = 0.112	*p* < 0.0001

CIS, Clinically isolated syndrome; BMI, body mass index; EDSS, expanded disability status scale; IFN-β, interferon beta. The Kruskal-Wallis test with Dunn’s multiple comparisons was used when comparing three groups. The Mann-Whitney test was used to compare the two groups. The Fisher’s exact test was used to compare the statistical differences in sex between groups.

Samples from persons with multiple sclerosis were collected at baseline, and after every 3 months up to one year (newly diagnosed, treatment naive PwMS) or 24 months (PwMS with relapse under IFN-β treatment) of ocrelizumab treatment, samples from healthy controls were collected at a single time point. Samples were stored at −80°C until analysis.

### Method details

#### Detection of serum biomarkers

The analyzed biomarkers related to gut barrier function and inflammatory response were quantified in serum using a commercial enzyme-linked immunosorbent assay (ELISA).

**Table:** The quantified biomarkers in sera

Biomarker	Abbreviation	Manufacturer	Cat. no.
CCL17/TARC	CCL17	R&D systems	DY364
Interleukin-18	IL-18	R&D systems	DY318-05
Mannose-Binding Lectin	MBL	R&D systems	DY2307
Lipopolysaccharide-Binding Protein	LBP	R&D systems	DY870
Soluble CD14	CD14	R&D systems	DY383
Tissue Inhibitor of Metalloproteinase 1	TIMP-1	R&D systems	DY970

#### Preparation of bacterial antigens and detection of specific antibodies

We selected typical representatives of a healthy Czech gut microbiota based on data from our previous study.^16^ The different bacteria were cultivated in their respective optimal media for 24 h at 37 °C (Table S1). As previously published, fresh bacterial cultures were centrifuged, and the resulting pellets were washed in sterile water and inactivated using a French press (French Pressure Cell Press model FA-078, SLM Instruments) at 1,500 PSIG, the pressing procedure was repeated three times. Samples were lyophilized in a freeze dryer (Lyovac GT 2, Leybold Heraeus) and stored in aliquots at −20 °C until analyzed. The following bacterial strains were used for antigen coating in the indirect ELISA: *Lactobacillus plantarum*, *Bifidobacterium adolescentis*, *Blautia coccoides*, *Roseburia intestinalis*, *Eubacterium rectale*, *Faecalibacterium prausnitzii*, *Ruminococcus flavefaciens*, *Bacteroides thetaiotaomicron*, *Prevotella ruminicola* and *Escherichia coli*. Indirect ELISA was performed as we described previously.^88^

#### Gut microbiota analysis

Analysis of gut microbiota from stool samples was done as previously described.^87^ DNA was isolated from the stool specimens using ZymoBIOMICS DNA Miniprep Kits (Zymo Research, Irvine, CA, USA) according to the manufacturer’s protocol. The V3 and V4 regions of the bacterial 16S rRNA genes were amplified using specific primers with barcodes (341F GTCCTACGGGNGGCWGCAG and 806R GGACTACHVGGGTWTCTAAT), respectively. V3 and V4 were chosen as representative sequences for taxonomic identification. The amplification reaction was performed using KAPA HiFi HotStart Ready Mix (Roche, Penzberg, Germany), as follows: the initial denaturation step for 3 min at 95 °C followed by 25 cycles at 95 °C for 30 s, 55 °C for 30 s, 72 °C for 30 s and a final elongation step at 72 °C for 5 min using 5 ng/μL DNA. PCR products were checked using QIAxcel advanced capillary electrophoresis (QIAgen, Hilden, Germany). Triplicates of the amplicons were pooled, normalized with SequalPrep Normalization Plate Kit (Thermo Fisher Scientific, Waltham, MA, USA), concentrated (Eppendorf centrifugal vacuum concentrator), and purified using DNA Clean & Concentrator Kit (Zymo Research). Subsequently, the amplicon libraries were ligated with sequencing adapters using KAPA HyperPlus Kit (Roche), pooled in equimolar concentrations, and sequenced. Amplicon sequencing was performed using the Miseq platform (Illumina, San Diego, CA, USA).

#### Analysis of virome

Plasma and stool samples from newly diagnosed PwMS (M0) treated with ocrelizumab for 3 (M3) or 6 months (M6), and from healthy controls (HC M0) living in the same household, were sequenced using the NetoVir protocol.^89^^,^^90^ The protocol was slightly modified for plasma samples (see below). Only samples with sufficient material that had not been thawed before were selected for virome analysis. In total, we analyzed 32 samples. Virome composition of 7 plasma samples of PwMS and their household controls was analyzed at M0. Of these patients three were also sampled at M3 and at M6. Virome composition of in stool was analyzed in five newly diagnosed PwMS and two household controls. Of these two patients were sampled at M3 and three patients at M6. To control laboratory and environmental contamination, a 1xPBS was used as the negative control and processed along with patient’s plasma and stool samples. To enrich virus-like particles (VLPs), 260 μL of plasma and the 1× PBS as a negative control were centrifuged for 3 min at 17,000 × g and filtered through 0.8 μm polyether sulphone filters (Sartorius, Göttingen, Germany). Nucleic acids not protected inside VLPs were removed by nuclease treatment with micrococcal nuclease (New England Biolabs, Ipswich, MA, United States) and benzonase (Millipore, Burlington, United States) and extracted using QIAamp viral RNA mini kit (Qiagen, Hilden, Germany). Pre-amplification was performed using WTA 2 kit (Sigma-Aldrich, St. Louis, MO, United States), and libraries were prepared for sequencing by using Nextera XT kit (Illumina, San Diego, CA, United States). The quality, size and concentration of these libraries were evaluated on a Bioanalyzer 2100 instrument (Agilent Technologies, CA, United States) and a Qubit 2.0 fluorometer (Thermo Fisher Scientific, Waltham, MA, United States), pooled in equimolar concentrations, and sequenced on a NextSeq 2000 platform (Illumina, San Diego, CA, United States) for 300 cycles (2 × 150 bp paired-ends), with a minimum of 10M reads per sample.

### Quantification and statistical analysis

#### Sequencing data processing and statistics for bacterial analysis

In the first step, sample demultiplexing, primer detection, and trimming were performed using Skewer.^91^ Low-quality reads (expected error rate per paired-end read >2) were then eliminated. DADA2 was used to denoise the quality-filtered reads and quantify 16S rRNA Amplicon Sequence Variants (hereafter, ASVs) in each sample.^92^ Chimeric ASVs were detected and eliminated using UCHIME and the gold.fna reference database (https://drive5.com/uchime/gold.fa).^93^ Taxonomical assignment of non-chimeric ASVs was conducted using the RDP classifier with an 80% confidence threshold and the Silva database (release 138) as a Quast et al^94^ Chloroplasts and mitochondria, as well as sequences that could not be assigned to a bacterial phylum, were considered contaminants of the diet or sequencing artifacts and were excluded from all downstream analyses. Sequences from technical duplicates were merged for each sample. The ASV abundance matrix (i.e., the number of ASV reads in each sample), ASV sequences, their taxonomic annotations, and phylogeny were merged into a single database along with sample metadata using the phyloseq package in R (R Core Team 2020).^95^ Sequencing data are available in the European Nucleotide Archive (ENA). To standardize sequencing depth, we rarefied the ASV table for alpha and beta diversity analysis. Rarefaction depth was set to the sample size of the minimal sequencing depth, i.e., where the majority of species were observed within a given number of samples. The alpha diversity was expressed as the Shannon diversity index, observed taxa ASVs, and Chao1 and compared using the Kruskal-Wallis test, followed by Dunn’s test for multiple comparisons or using the Wilcoxon matched-pairs signed rank test for paired samples between M0 and M12 according to treatment response Principal Coordinate Analysis (PCoA) was performed to investigate differences in microbiota composition between samples. We also used relative ASV abundance (Bray-Curtis index; Bray and Curtis 1957).^96^^,^^97^ To evaluate the differences in beta diversity between groups, we used the Permutational Multivariate Analysis of Variance Using Distance Matrices (PERMANOVA) with a vegan package.^98^ Taxonomical analysis was performed using the microViz package, and relative abundances were calculated and presented.^99^ Associations between clinical metadata and microbial features were tested in R using the MaAsLin2 package after TSS normalization and LOG transformation of data, the *p*-values were corrected using Benjamini–Hochberg false-discovery-rate (FDR) correction.^100^ The output is depicted as standard MaAsLin2 output as a heatmap with significantly increased (+) or decreased (−) bacteria as compared to group specified in the figure legend (e.g., HC or M0). The Phylogenetic Investigation of Communities by Reconstruction of Unobserved States (PICTRUs2) tool was used to predict microbiome function with 16S rRNA gene data.^101^ The downstream analysis of data was performed using the ggpicrust2 R package.^102^ The ggplot2 package was used for graphical visualization of the data.^103^ Microbiome analysis sequencing data are available in the European Nucleotide Archive (ENA) (https://www.ebi.ac.uk/ena/browser/home) under accession number PRJEB83221.

#### Sequencing data processing and statistics for virome analysis

The quality of reads was checked using FastQC (Babraham Bioinformatics, Cambridge, UK) both before and after trimming sequences to remove WTA2, Nextera adapters, and low-quality reads/bases, which was done using Trimmomatic v0.39.^104^ After QC and trimming, we gain an average of about 10M reads per sample (from 8.5M to 15M). The reads were assembled with SPAdes v3.15.5 with k-mers k = 21, 33, 55, and 77.^105^ The assembled contigs were blasted against the non-redundant protein NCBI database (downloaded on 24.1.2024) with DIAMOND v2.1.8^106^ and subsequently visualized using Kronatools.^107^ Contigs were pooled from all the analyzed samples, limited to those >500 bp, and dereplicated (95% identity over 85% of sequence). The resulting non-redundant contigs were blasted with DIAMOND against the nr protein database but with flags (sensitive and -c1), and then reads from each sample were mapped on these contigs using bwa-mem2 v2.2.1.^108^ Reads mappings were extracted with CoverM v0.6.1 (https://github.com/wwood/CoverM). Subsequent analysis was done in R using the phyloseq package. Sequencing data are available in the NCBI under bioproject PRJNA1194976.

#### Analysis of viral composition

The obtained data were imported into R and converted to a phyloseq object using Phyloseq package.^95^ Data were normalized at the same sequencing depth. A QC of the resulting data was done at this point (NC- mapping on the set of contigs; unusual viruses that might be misclassified) and taxonomy was curated into groups. The alpha diversity and PCoA was calculated with a microbiome package (Shannon diversity measure).^109^ The differentially abundant taxa were identified with DESeq2 (p adj cutoff <0.05, log2fc cutoff <1) and heatmap with differentially abundant viral contigs from comparison of HC vs. M0, and M0 vs. later time points was created with a pheatmap package.^110^ The boxplot of relative abundance was created with a ggplot2 package.^103^ The significance was done with ggpubr package^111^ using Mann Whitney U test, *p*-values were adjusted for multiple testing with Benjamini-Hochberg FDR method.

#### Statistical analysis of antibodies and biomarkers

Interconnections between molecules were shown using the protein-protein network functional enrichment analysis based on co-expression, cooccurrence, experimentally determined, and both text mining and curated databases using the STRING database and STRING Consortium 2023 web source.^112^

The non-parametric Kruskal–Wallis test with Dunn’s multiple comparisons test (at a single time point, e.g., HC vs. all time points) and the Friedman test with Dunn’s multiple comparisons test (in longitudinal data analysis, e.g., M0 vs. M6 and M12) were used to compare multiple experimental groups. The non-parametric Mann–Whitney test was used to compare two experimental groups, and the Wilcoxon matched-pairs signed rank test was used to compare paired data inside one group. The data are presented as medians with 95% confidence interval (CI) (if not stated otherwise). Differences were considered statistically significant at *p* ≤ 0.05 unless otherwise stated. GraphPad Prism statistical software (version 8.1.1, GraphPad Software, San Diego, CA, USA) was used for statistical analyses. Other analyses were performed on the R statistics platform (R Foundation for Statistical Computing, http://www.r-project.org).^113^

## Supplemental information

Document S1. Figures S1–S11

Table S1. Bacteria and cultivation conditions
