# Supplementary material for: Ocrelizumab transiently alters microbiota and modulates immune response depending on treatment outcome
Source: iScience. 2025 Oct 31;28(12):113872. doi: 10.1016/j.isci.2025.113872 (PMC12677075; doi:10.1016/j.isci.2025.113872)
Supplement: Document S1. Figures S1–S11 [file mmc1.pdf]

**iScience, Volume *28***

## Supplemental information

# Ocrelizumab transiently alters microbiota and modulates immune response depending on treatment outcome

**Stepan Coufal, Zuzana Jiraskova Zakostelska, Tomas Thon, Radka Roubalova, Dominika Kadleckova, Martina Salakova, Ruth Tachezy, Tomas Hrncir, Miloslav Kverka, Veronika Ticha, Miluse Pavelcova, Pavlina Kleinova, Jana Lizrova Preiningerova, Ivana Kovarova, Jakub Kreisinger, Helena Tlaskalova-Hogenova, and Eva Kubala Havrdova**

## Supplementary materials

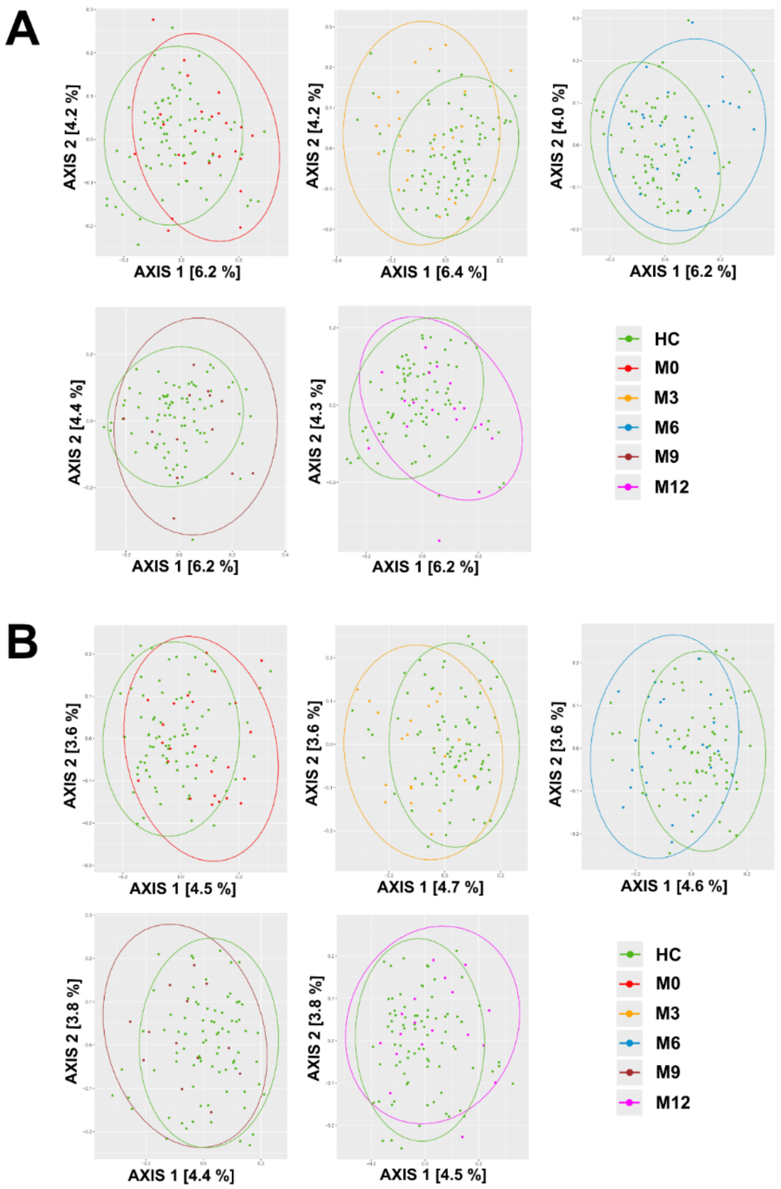


**Figure S1: Individual comparison of beta diversity between HC and newly diagnosed PwMS undergoing treatment with ocrelizumab. Related to Figure 1B.** Individual comparison of beta diversity is expressed as Bray Curtis (A) and Jaccard index (B) from Figure 1B. Compared using the Permutational Multivariate Analysis of Variance Using Distance Matrices (PERMANOVA) (HC n=81, M0 n=25, M3 n=23, M6 n=26, M9 n=13, M12 n=18).

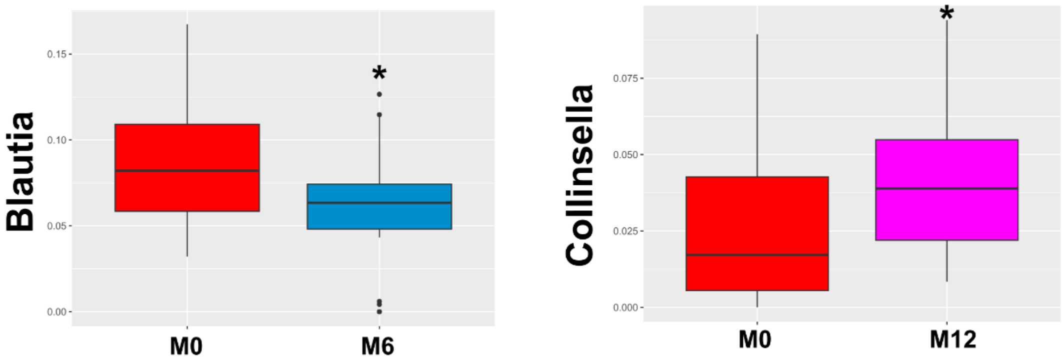


**Figure S2: Detailed analysis of the data depicted in Figure 1E. Significant association of genus *Blautia* and *Collinsella* with treatment duration (months). Related to Figure 1E.** Compared to M0 using the MaAsLin2 R package (M0 n=25, M12 n=18). Data are presented as median; ∗ ($p < 0.05$).

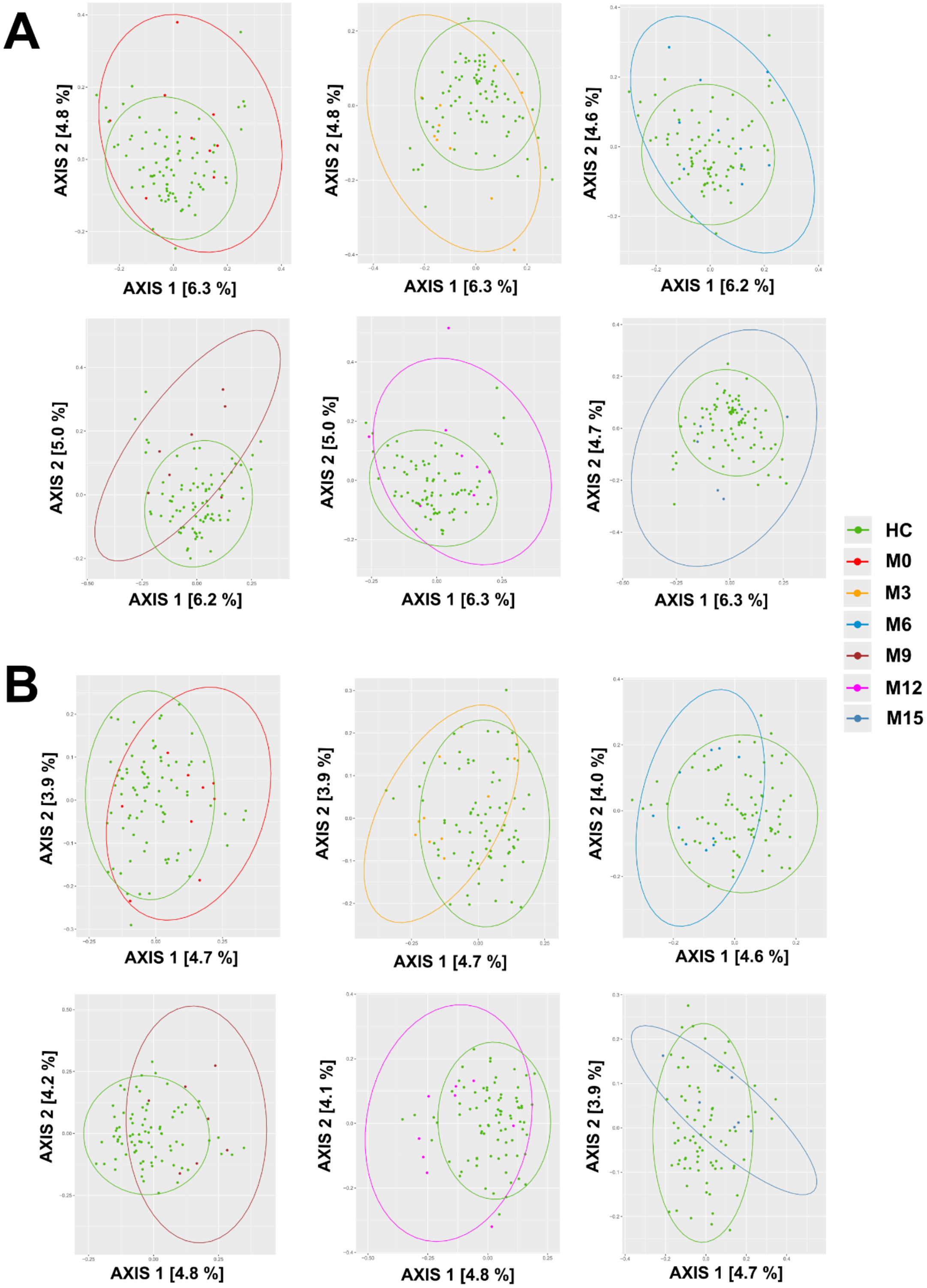


**Figure S3: Individual comparison of beta diversity of PwMS with interferon beta treatment failure undergoing treatment with ocrelizumab. Related to Figure 2B.** Individual comparison of beta diversity is expressed as Bray Curtis (A) and Jaccard index (B) from Figure 2B. Compared using the Permutational Multivariate Analysis of Variance Using Distance Matrices (PERMANOVA) (HC n=81, M0 n=9, M3 n=10, M6 n=10, M9 n=7, M12 n=9, M15 n=6).

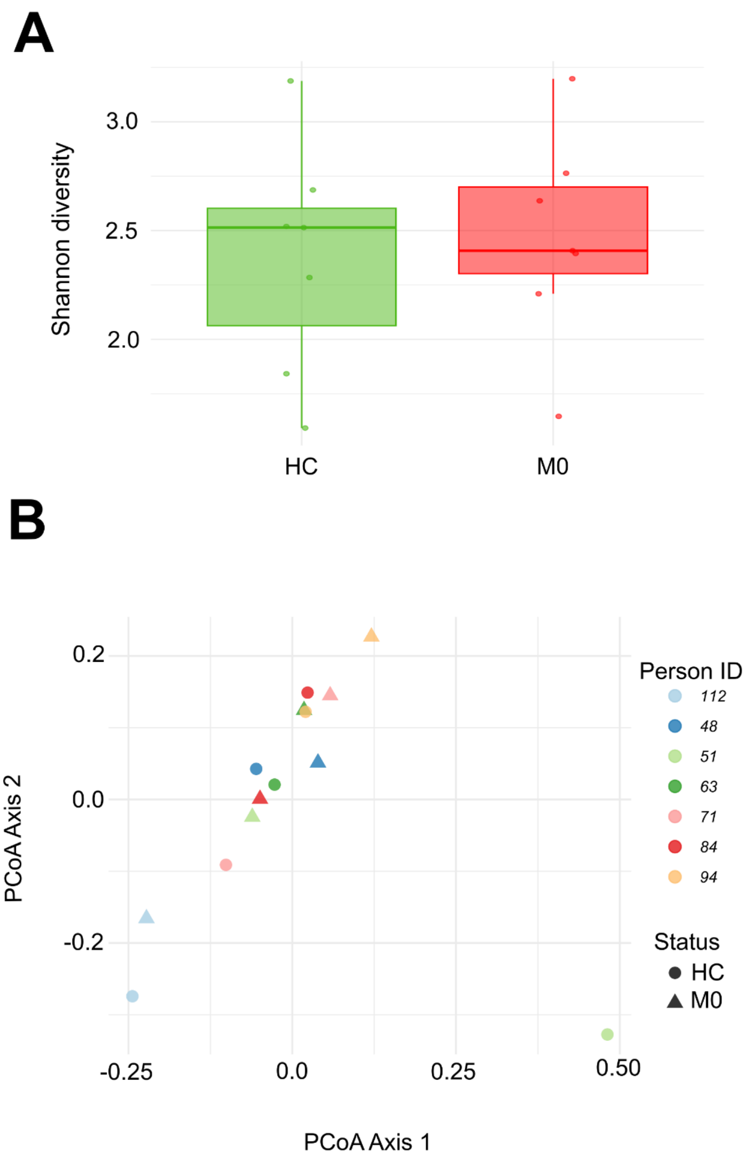


**Figure S4: Plasma virome alpha and beta diversity in newly diagnosed PwMS and HC households. Related to Figure 3.** Alpha diversity of eukaryotic viruses; data are presented as median (A). Beta diversity (B). (M0 n=7, HC n=7).

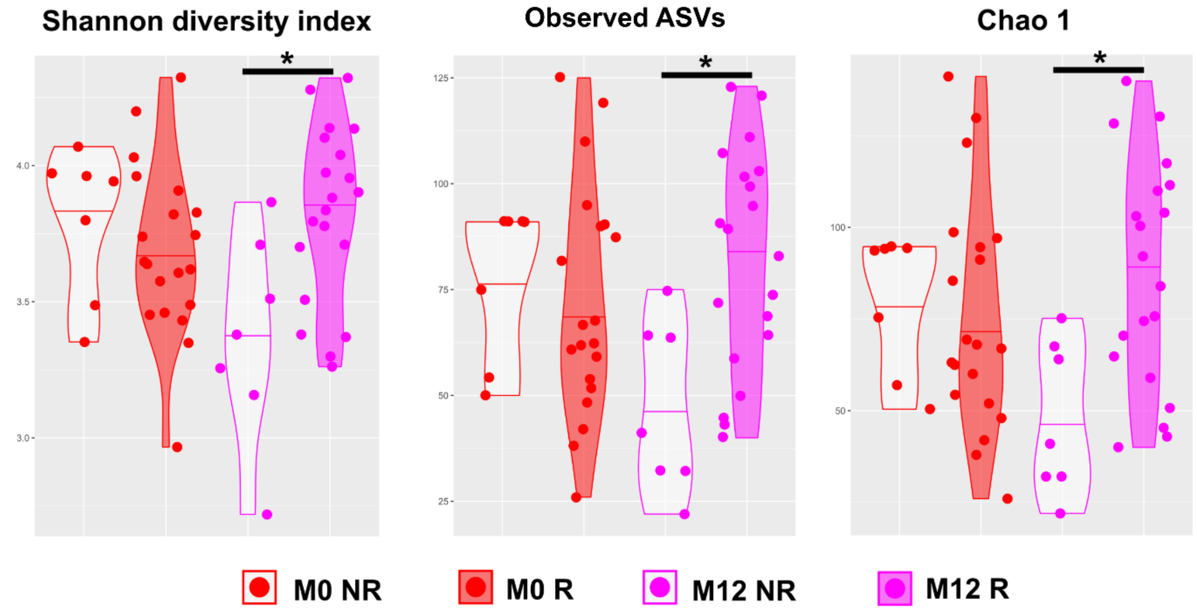


**Figure S5: Differences in alpha diversity according to the treatment response. Related to Figure 4A.** Alpha diversity is expressed as the Shannon diversity index, Observed ASVs, and Chao1 (A). Data are presented as median. * ($p < 0.05$) is used to depict significant differences compared between groups using the Kruskal-Wallis test with Dunn's multiple comparisons test (M0 NR n=7, M12 NR n=7, M0 R n=20, M12 R n=20); ∗ ($p < 0.05$).

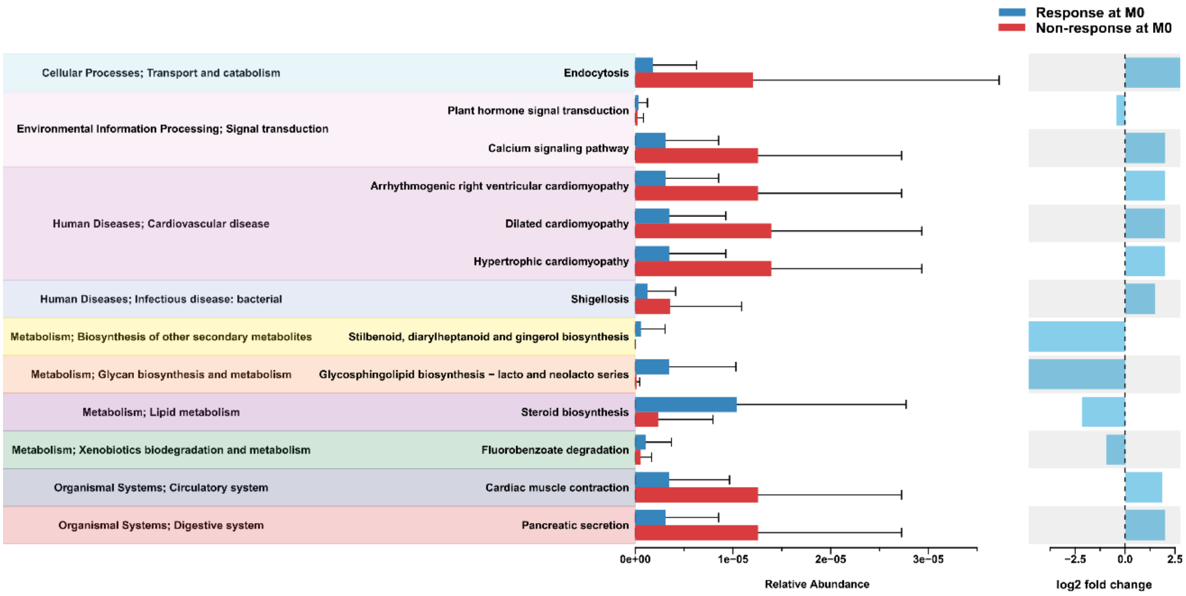


**Figure S6: Differential abundance of functional pathways in the microbiota of responders and non-responders at M0.** Only significant pathways with adjusted $p < 0.05$ are depicted after adjustment; mean and standard deviation are shown (Response n=20, Non-response n=7).

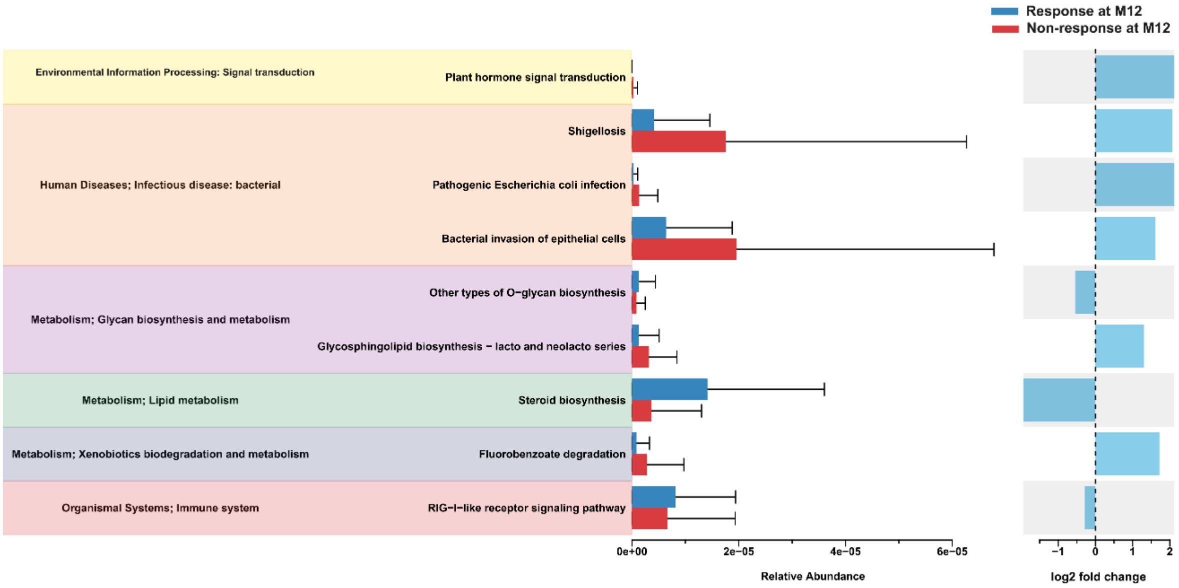


**Figure S7: Differential abundance of functional pathways in the microbiota of responders and non-responders at the end of M12.** Only significant pathways with adjusted $p < 0.05$ are depicted after adjustment; mean and standard deviation are shown (Response n=20, Non-response n=7).

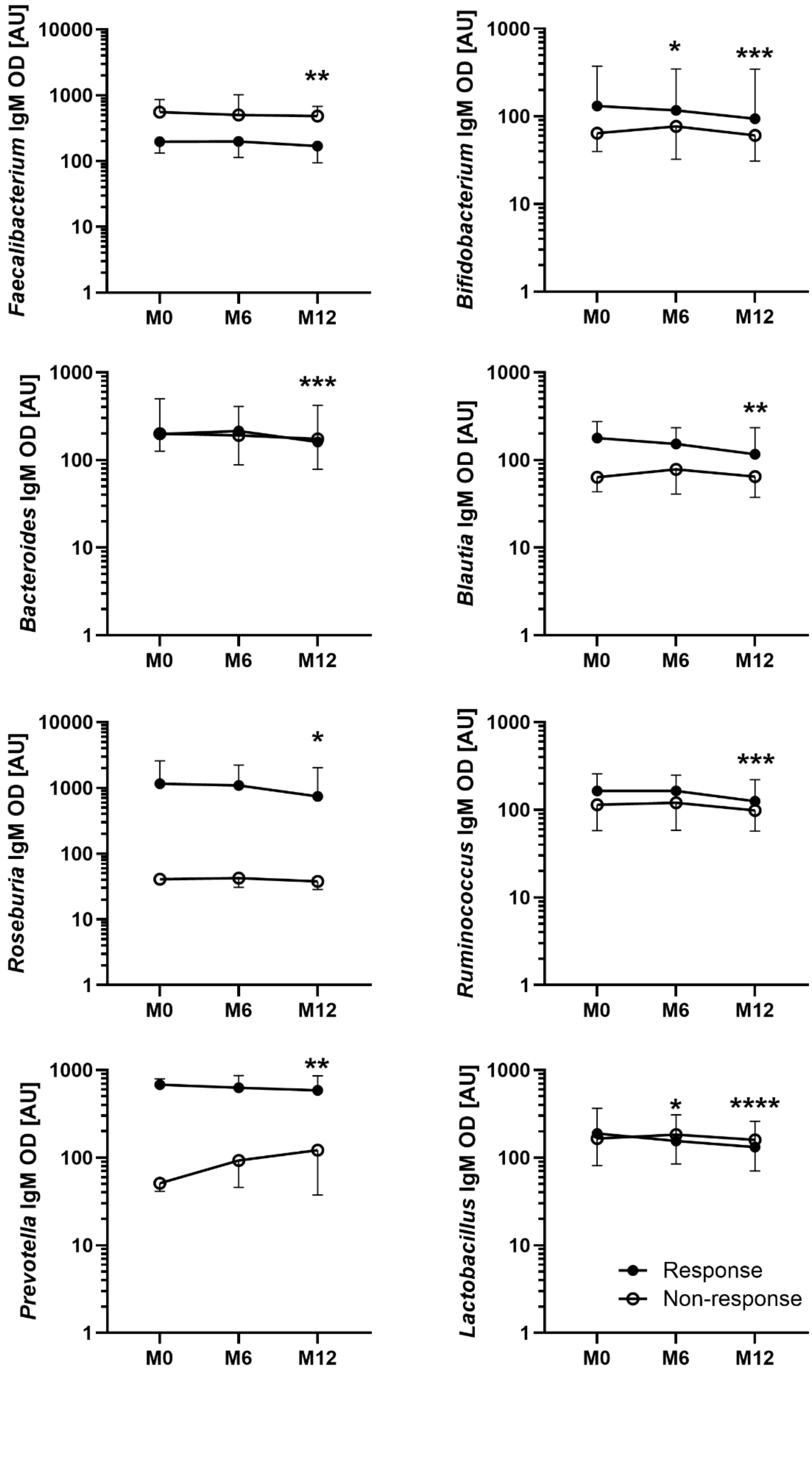


**Figure S8: Anti-commensal IgM antibodies according to the treatment response.** The Friedman test with Dunn's multiple comparisons test was used to compare statistical differences between time points within groups. Data are presented as median + 95% CI. * depicts a significant decrease after 6 or 12 months of treatment in responders. (Responders n=12, Non-responders n=4); ∗ ($p$ < 0.05), ∗∗ ($p$ < 0.01), ∗∗∗ ($p$ < 0.01), ∗∗∗∗ ($p$ < 0.001).

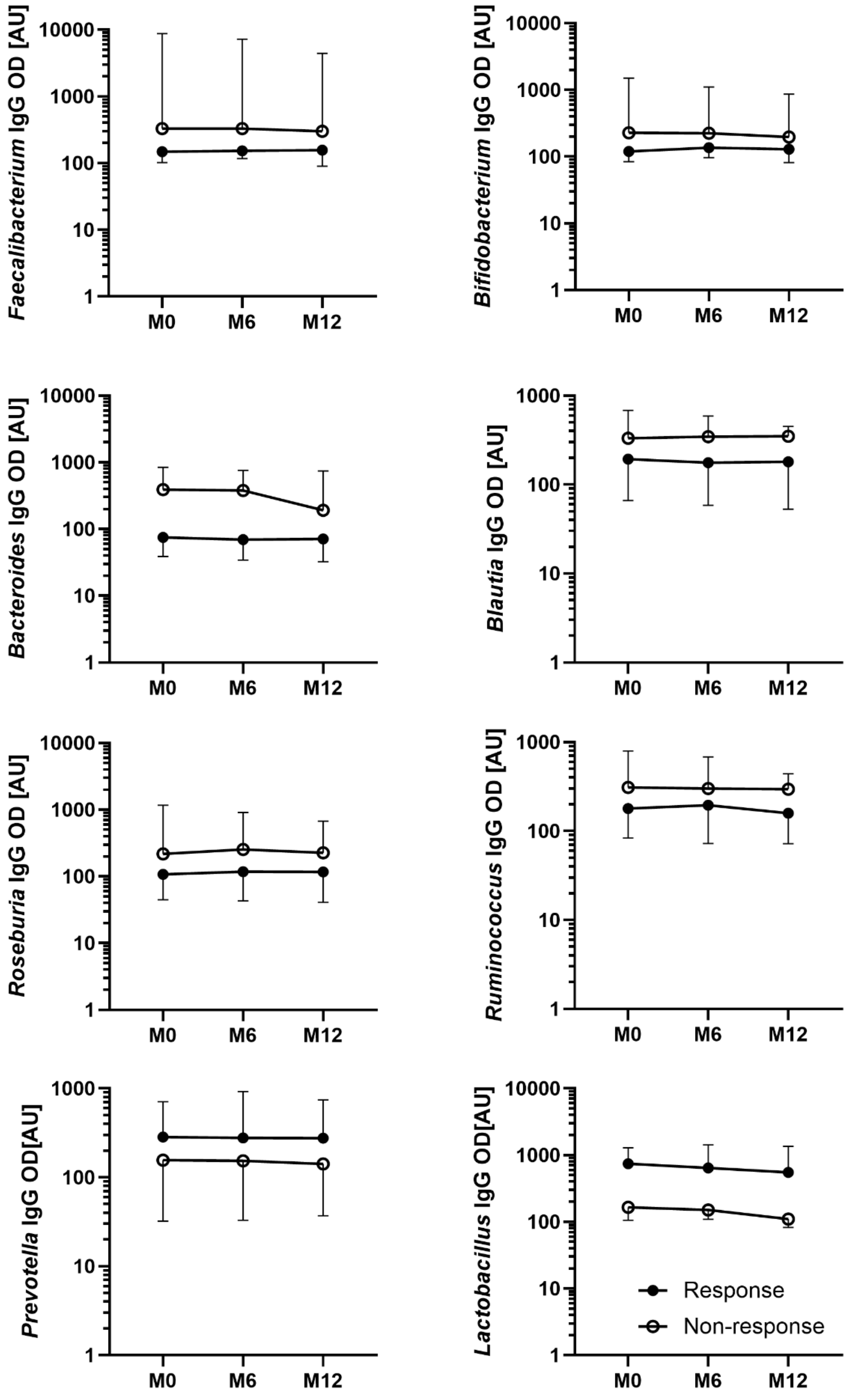


**Figure S9: Anti-commensal IgG antibodies according to the treatment response.** Data are presented as median + 95% CI. (Responders n=12, Non-responders n=4).

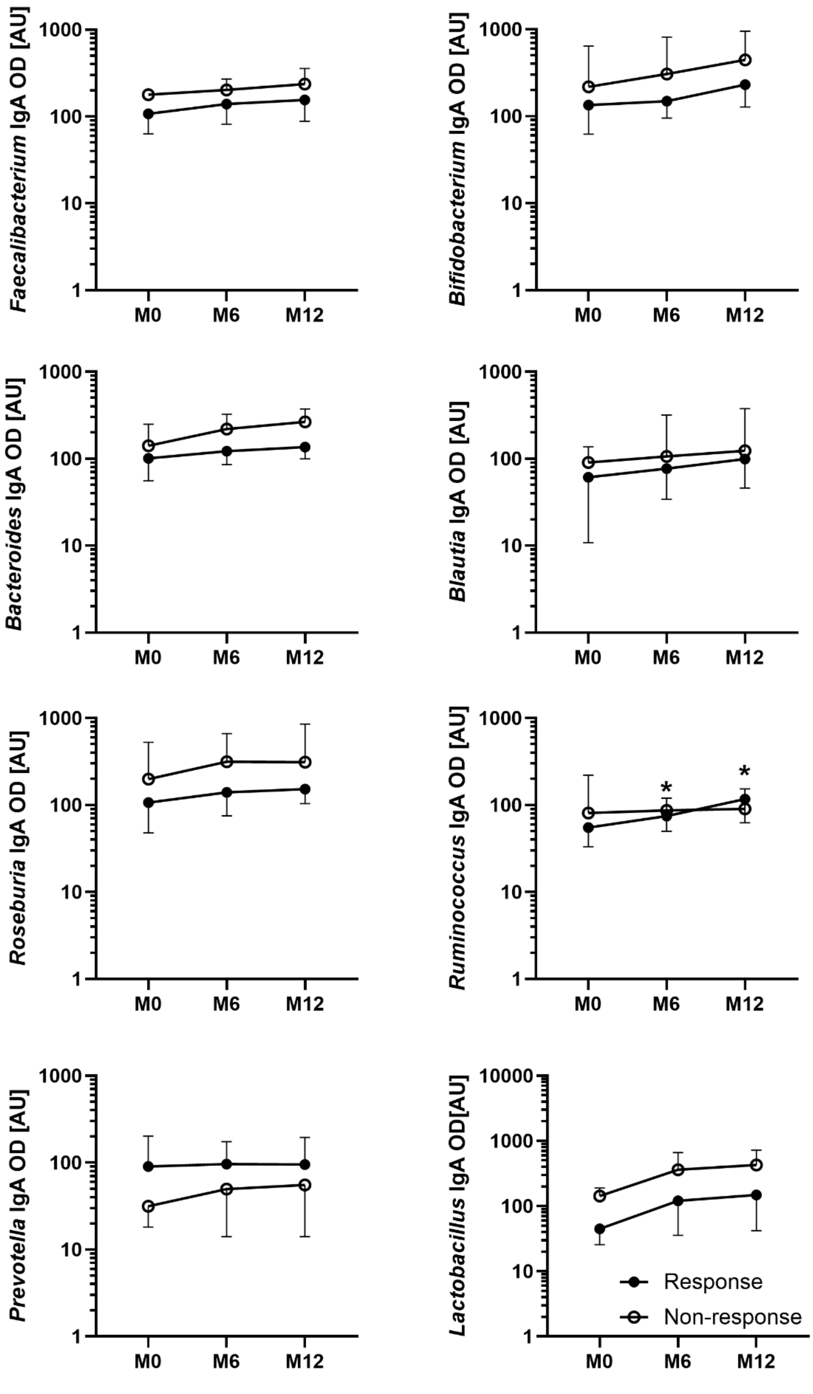


**Figure S10: Anti-commensal IgA antibodies according to the treatment response.** The Friedman test with Dunn's multiple comparisons test was used to compare statistical differences between time points within groups. Data are presented as median + 95% CI. * depicts a significant increase after 6 or 12 months of treatment in responders. (Responders n=12, Non-responders n=4).

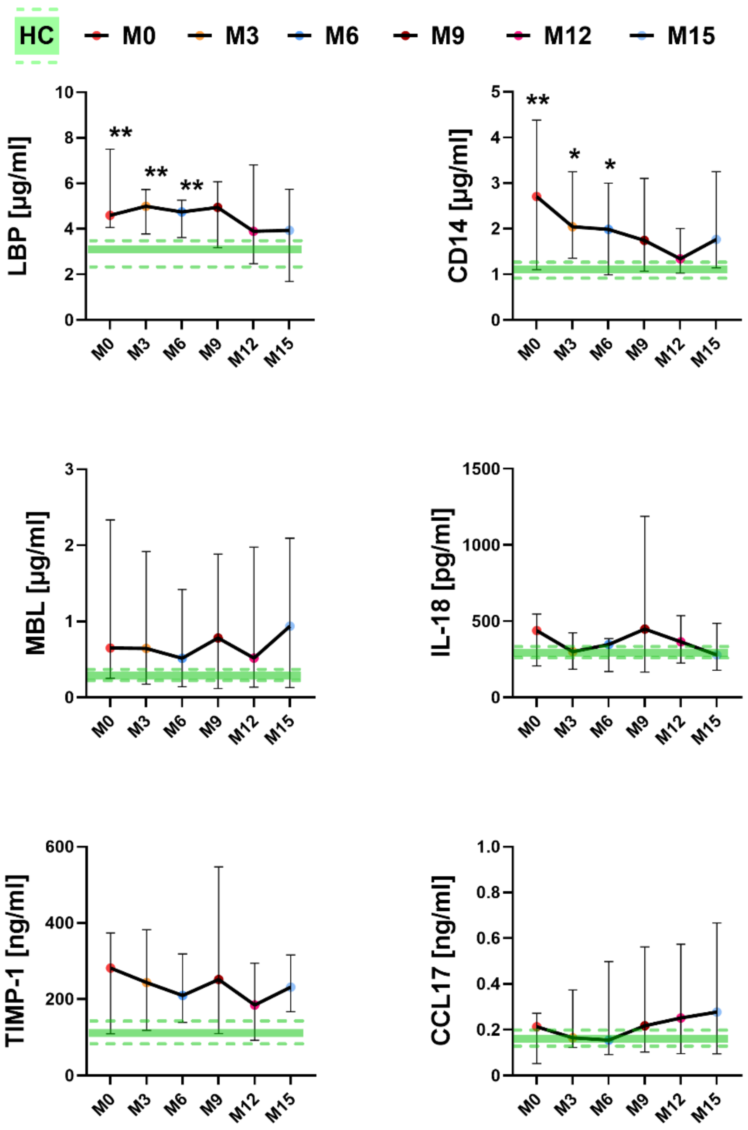


**Figure S11: Levels of serum biomarkers in PwMS with interferon beta treatment failure undergoing treatment with ocrelizumab.** Lipopolysaccharide-binding protein (LBP), soluble CD14 (CD14), Mannose-binding lectin (MBL), Tissue inhibitor of metalloproteinase – 1 (TIMP-1), Interleukin-18 (IL-18), C-C motif chemokine ligand 17 (CCL17). Data are presented as median + 95% CI. Green lines depict median + 95% CI of HC. The Kruskal-Wallis test with Dunn's multiple comparisons test was used to compare all time points to HC. * depicts significant differences at single time points in MS patients compared to HC. (HC n=61, M0 n=9, M3 n=10, M6 n=10, M9 n=6, M12 n=7, M15 n=6); ∗ ($p$ < 0.05), ∗∗ ($p$ < 0.01).
