# Supplementary material for: Ocrelizumab transiently alters microbiota and modulates immune response depending on treatment outcome
Source: iScience. 2025 Oct 31;28(12):113872. doi: 10.1016/j.isci.2025.113872 (PMC12677075; doi:10.1016/j.isci.2025.113872)
Supplement: Table S1. Bacteria and cultivation conditions [file mmc2.pdf]

Supplementary Materials:

Bacterium	Cultivation medium	Cultivation condition
Lactobacillus plantarum CCDM 182	MRS Broth for Lactobacilli (ATCC Medium No. 416)	anaerobic
Bifidobacterium adolescentis CCUG 18363	DSMZ medium No. 58. for Bifidobacteria	anaerobic
Blautia coccoides	Modified chopped meat medium (ATCC Medium No. 1490)	anaerobic
Roseburia intestinalis L1-82	Keister's Modified TYI-S-33 (ATCC Medium No. 2695)	anaerobic
Eubacterium rectale ATCC 33656	Chopped meat carbohydrates with rumen fluid (ATCC Medium No 1703)	anaerobic
Faecalibacterium prausnitzii A2-165	Modified YCFA medium (DSMZ medium No. 1611)	anaerobic
Ruminococcus flavefaciens DSM 25089	Medium for anerobes with 0.1% cellobiose (ATCC Medium No. 1365 E)	anaerobic
Bacteroides thetaiotaomicron VPI 5482	Modified chopped meat medium (ATCC Medium No. 1490)	anaerobic
Prevotella ruminicola M384	Chopped meat carbohydrates with rumen fluid (ATCC Medium No 1703)	anaerobic
Escherichia coli K6	Luria-Bertani broth (Merck, L3022)	aerobic

Table S1: Bacteria and cultivation conditions
